# eBEfree: Combining Psychoeducation, Mindfulness, and Self‐Compassion in an App‐Based Psychological Intervention to Manage Binge‐Eating Symptoms: A Randomized Controlled Trial

**DOI:** 10.1002/eat.24432

**Published:** 2025-03-29

**Authors:** Hugo Senra, Cristiana Duarte, Sérgio A. Carvalho, Luís Simões, Cláudia Ferreira, Lara Palmeira, Marcela Matos, Marina Cunha, Paula Castilho, Bruno Sousa, Luis Cordeiro, José Pinto‐Gouveia

**Affiliations:** ^1^ Institute of Electronics and Informatics Engineering of Aveiro (IEETA) University of Aveiro Aveiro Portugal; ^2^ School of Health and Social Care University of Essex Essex UK; ^3^ School of Education, Language and Psychology York St. John University York UK; ^4^ University of Coimbra, Center for Research in Neuropsychology and Cognitive and Behavioral Intervention (CINEICC) Coimbra Portugal; ^5^ Coimbra Hospital and University Centre (CHUC) Coimbra Portugal; ^6^ CINTESIS@RISE, CINTESIS.UPT Portucalense University Porto Portugal; ^7^ Instituto Superior Miguel Torga Coimbra Portugal; ^8^ OneSource Consulting Coimbra Portugal

**Keywords:** acceptance and commitment therapy, binge‐eating, cognitive‐behavioral therapy, depression, eating disorders, mindfulness, randomized clinical trial, self‐compassion

## Abstract

**Objective:**

To develop and test the effectiveness of the eBEfree program, a 12‐session app‐based version of a previously tested psychological intervention (BEfree) that combines psycho‐education, self‐compassion, mindfulness, and Acceptance and Commitment Therapy to reduce binge‐eating symptoms.

**Method:**

Two‐hundred participants with recurrent elevated binge‐eating symptoms and a higher body weight were enrolled in a remote parallel‐group randomized trial, of which 142 completed the initial assessment (70 in the intervention group). The end‐of‐treatment and follow‐up assessments were conducted 12 weeks after the initial assessment and 26 weeks after the end of treatment, respectively. Intention‐to‐treat (ITT) analyzes were undertaken using frequentist linear mixed models and Bayesian hierarchical models to test the intervention effectiveness.

**Results:**

43 (waitlist control) and 29 (intervention) participants completed the end‐of‐treatment assessment, showing a high attrition rate (51%). ITT analyzes showed the eBEfree program to be associated with a significant reduction in binge eating symptomatology from baseline to end‐of‐treatment (*β* = −9.49, *ρ* < 0.0001, *g* = −1.17), and from end‐of‐treatment to 26‐weeks follow‐up (*β* = −6.01, *ρ* = 0.04, *g* = −1.08). At end‐of‐treatment, the intervention was also associated with a decrease in body mass index and depression symptomatology, as well as an improvement in dimensions of self‐criticism and mindfulness. More than 80% of participants rated the quality of the program as positive and helpful.

**Discussion:**

This trial suggests potential benefits of the eBEfree program to help individuals manage symptoms of binge‐eating more effectively, reduce weight, and improve well‐being and mental health. The intervention benefits should be confirmed in future larger trials.

**Public Significance:**

Treating binge‐eating (BE) symptoms and associated comorbidities might be challenging for mental health services. Digital and app‐based interventions can be a valuable resource to help people manage symptoms and improve well‐being and mental health. The current trial suggests the potential benefits of an app‐based intervention (eBEfree program) to help people reduce BE symptoms and develop psychological strategies to promote well‐being and mental health.

**Trial Registration:** Clinicaltrials.gov: NCT04101032 (“eBEfree—an ICT Adaptation of BEfree”)

## 1 Introduction

Binge‐eating disorder (BED) is a potentially disabling condition entailing negative consequences for health, psychological, and social functioning (di Giacomo et al. 2022; Giel et al. 2022; Keski‐Rahkonen 2021; Mourilhe et al. 2021). Although BED is common in individuals with a healthy body weight, it has also been associated with a high risk for long‐term overweight and obesity (Keski‐Rahkonen 2021; Santomauro et al. 2021; Udo and Grilo 2018). A large population‐based study estimated a mean body mass index of 34.9 in individuals with a 12‐month BED diagnosis (Udo and Grilo 2018). In a 10‐year prospective study, eating disorders, including binge eating, were associated with a threefold increase in lifetime obesity, with individuals with binge eating showing the highest rates of obesity (88%) (Villarejo et al. 2012). Previous research has also highlighted the elevated co‐occurrence of binge‐eating symptoms in individuals seeking weight loss treatment and in individuals at the pre‐operative stage of bariatric surgery (Ágh et al. 2015; Dawes et al. 2016).

Recent meta‐analyzes have highlighted heterogeneous results of treatment effectiveness in individuals with elevated binge eating symptomatology (Hilbert et al. 2019, 2020). The effect sizes are moderate to high (in comparison with inactive controls) in pharmacological treatments, mainly testing second‐generation anti‐depressants (0.45, [95% CI: 0.34–0.57]), and in psychological treatments, mainly testing cognitive‐behavioral therapy (0.83 [95% CI: 0.45–1.2]) for reducing binge‐eating episodes. However, the effect sizes get considerably lower when examining the treatments' effects on reducing body mass index, both at the end‐of‐treatment and follow‐up analysis (Hilbert et al. 2019, 2020), with the same meta‐analyzes reporting an overall low study quality and unclear/low level of evidence, due to studies' limitations, inconsistencies, indirectness, imprecision, and publication bias (Hilbert et al. 2019, 2020).

Among psychological treatments tested for BED, cognitive‐behavioral based therapies have shown promising results to reduce binge‐eating symptoms and to improve mental health (e.g., reducing eating disorder psychopathology and depression) (Grilo et al. 2020; Hilbert et al. 2019, 2020; Linardon et al. 2017). Other psychological interventions such as mindfulness‐based and compassion‐focused therapies have also shown potential for reducing binge eating behaviors and comorbid mental health problems (Duarte et al. 2021; Grohmann and Laws 2021; Katterman et al. 2014; Kelly and Carter 2015; Messer et al. 2021; Sala et al. 2020; Serpell et al. 2020; Warren et al. 2017). A randomized trial found an association between self‐compassion strategies developed through an 8‐week mindful self‐compassion group intervention and an improvement in negative affect linked to binge‐eating behavior and also an improvement in food‐related self‐regulation and calorie intake (Serpell et al. 2020). A large‐scale interventional study suggested the potential benefit of a compassion‐based intervention to reduce binge‐eating symptoms and to improve psychological adjustment and mental health (Duarte et al. 2021). The study found compassion, self‐reassurance, and a reduction in self‐criticism to mediate the positive effect of the compassion‐focused intervention on binge‐eating behaviors.

Mindfulness‐based interventions have been tested on individuals with elevated binge‐eating symptoms (Grohmann and Laws 2021; Katterman et al. 2014). A meta‐analysis examining 11 randomized controlled trials highlighted the potential benefit of mindfulness‐based interventions for reducing binge eating severity (Hedge's *g* = −0.39; 95% CI: −0.68, −0.11), although the significant effect was only seen at the end of treatment, with no maintained effect at follow‐up (Grohmann and Laws 2021). Improvements in depression symptomatology, emotion regulation, emotional eating, and binge‐eating behaviors have also been found in mindfulness‐based interventions, including in psychological therapies with a mindfulness component (Grohmann and Laws 2021; Warren et al. 2017).

Intervention programs combining different therapeutic approaches have also been tested for managing binge‐eating symptomatology. A randomized controlled trial tested a 12‐sessions group psychological intervention program for people with binge‐eating and overweight or obesity (BEfree program). This program incorporated different therapeutic approaches such as psycho‐education, self‐compassion, mindfulness, and Acceptance and Commitment Therapy (ACT) (Pinto‐Gouveia et al. 2019). Main end‐of‐treatment findings suggested a positive intervention effect on the reduction of BED symptoms (Cohen's *d* = 1.95), including in disordered eating (Cohen's *d* = 1.34) and also a reduction in body mass index (Cohen's *d* = 0.52). An improvement was seen in individuals' body‐image related symptoms, shame, self‐criticism, body‐image flexibility, self‐compassion, and general quality of life. The intervention‐related improvements were maintained at 3‐and 6‐month follow‐up. The BEfree program was designed to target key psychological and behavioral issues that have been previously identified to be closely associated with recurrent elevated binge eating symptomatology. This includes negative emotions and thoughts that are recurrently experienced by these individuals, triggering maladaptive eating behaviors such as binge eating (di Giacomo et al. 2022; Lee‐Winn et al. 2016; Wilson et al. 2021), lower cognitive control combined with higher negative affect (Vainik et al. 2019), poor emotion regulation (Svaldi et al. 2012), addictive behavior (di Giacomo et al. 2022), avoidance (Rotella et al. 2015), shame and negative body image (Norder et al. 2023; O'Loghlen et al. 2022).

The current study aims to extend the findings of the previous research that showed the effectiveness of an integrated group psychological intervention for managing binge eating symptomatology in individuals with overweight or obesity (BEfree program; Pinto‐Gouveia et al. 2019). The main goal is to develop and test the effectiveness of a digital version of the BEfree program, the eBEfree program, which is designed to tackle the significant gap in the uptake of mental health services among individuals with recurrent binge‐eating symptoms and a higher body weight (overweight and obesity), and to offer a potentially sustainable resource to address and manage binge‐eating symptomatology. As in the BEfree trial, the choice of a subsample of individuals with a higher body weight is justified by the elevated comorbidity of both conditions, making these individuals potentially more susceptible to complex health conditions (e.g., multimorbidity, obesity related diseases) (Giel et al. 2022; Keski‐Rahkonen 2021). In line with the BEfree trial, the eBEfree program was designed to incorporate different therapeutic approaches (Psycho‐education, self‐compassion, mindfulness, and acceptance and commitment therapy), as previous research had already suggested their individual potential to tackle psychological and behavioral issues related to BED (Hermanto and Zuroff 2016; Hill et al. 2015; Kelly and Carter 2015; Kristeller and Wolever 2010).

It is hypothesized that participants randomized to receive the intervention (eBEfree program) would present greater improvements in the primary outcome, binge‐eating symptoms, at end of treatment (12‐weeks after baseline) and at 26 weeks follow‐up. Additionally, it is also hypothesized that there would be an improvement in secondary outcomes associated with the eBEfree intervention at end of treatment and follow‐up stages, namely: body mass index; well‐being; mindfulness; self‐compassion; cognitive fusion in relation to body image (as part of the ACT component, which conceptualizes cognitive fusion as the tendency for over‐regulated and cognitively influenced behavior); acceptance (ACT component); shame; self‐criticism; depression symptomatology; and values‐based behavior related to the clarification of health‐related values and reflection on obstacles that have prevented living in accordance with those values.

## 2 Materials and Methods

### 2.1 Design

This study is a remote parallel‐group randomized trial comparing two groups of individuals with recurrent elevated binge eating symptoms and a higher body weight: (1) a group assigned to receive a 12‐session/week digital and app‐based mental health intervention based on the previously tested BEfree program (Pinto‐Gouveia et al. 2019), which comprises an integrative therapeutic approach combining psycho‐education, self‐compassion, mindfulness, and acceptance and commitment therapy; (2) a waitlist control group. Assessments were conducted at baseline, at 12 weeks post‐randomization (end‐of‐treatment), and 26 weeks after the end‐of‐treatment (follow‐up), to stick with the same trial assessment protocol adopted in the Befree trial (Pinto‐Gouveia et al. 2017). Participants completed online self‐report questionnaires (detailed below) which were already translated and validated to the Portuguese population. The original study design included a hybrid version of the trial, with participants being assigned for in‐person assessments and remotely delivered interventions. However, because this study was conducted during the SARS‐COV‐2 pandemic, we had to adjust the original trial protocol to make all trial procedures, including trial recruitment and assessment activities, run remotely. The current therapeutic program is called eBEfree, which is a digital adaptation of the previously tested BEfree program (Pinto‐Gouveia et al. 2019).

Prior to trial commencement, a pilot study was conducted with 10 participants, to check the adequacy and usability of our app‐based program, including the content (audios, videos, texts), and duration of session. The feedback collected from participants was taken to improve and finalize the final version of the app‐based program, ready to be delivered in the randomized trial.

The trial received ethical clearance from different institutional ethical committees, includingthe University of Coimbra and from the Regional Health Administration from the Portuguese National Health System (ref.: 98/2019), and was registered (ref.: NCT04101032). All participants were informed about all study procedures, includingblind randomization, online questionnaire, intervention format, content and duration, and gave their consent before any interview, assessment, or randomization procedure had taken place. The study was entirely conducted according to the Helsinki declaration and received informed consent from all participants.

### 2.2 Study Sample and Recruitment

Participants were recruited from February to July 2021 via public advertisements, including: University of Coimbra press releases; social media (weight management groups, obesity association groups, university social media pages); regional and national press and radio stations; and on the project website (https://ebefree.uc.pt/). On the website, we provided educational and more detailed content on the eBEfree program: what it is; for whom; how it was created (from BEfree program); what Binge Eating Disorder is (generic information); how the program works; how many sessions there are and their content; how to register interest to participate; and who we are (the research team). In the advertisement, potential participants were informed about basic eligibility criteria, which were adopted from the previous trial (BEfree program), such as: age (18–55); having overweight or obesity (body mass index equal or greater than 25); having access to a smartphone and willingness to use it; and not being enrolled in the BEfree trial (Pinto‐Gouveia et al. 2019).

Potential participants who registered their interest were first interviewed online by one of the eBEfree researchers who were also certified clinical psychologists. The online interviews were designed to meet the following goals: (1) to provide participants with a more detailed description of the eBEfree program, how it works, its content, duration, potential challenges, realistic goals, expectations, and assessment procedures; (2) to check participants' full eligibility to participate in the trial; and (3) if the participant was eligible, to discuss their motivation and expectations in relation to the program, including realistic goals and potential challenges during the program and main goals to be met at the end of the program.

Full inclusion criteria to participate in this trial included the basic inclusion criteria previously mentioned, plus: presence of elevated binge‐eating symptomatology; absence of severe depressive symptomatology, to avoid having participants with more complex mental health needs (e.g., suicide ideation), needing more immediate medical/psychological intervention that would interfere with our trial program; absence of other enduring mental health problems, such as personality disorders, and psychosis; and are not receiving other form of psychological or psychiatric intervention. Symptoms of binge‐eating were assessed by two qualified clinical psychologists using semi‐structured clinical interviews based on DSM‐5 criteria for BED (American Psychiatric Association 2013), and using the Binge‐Eating Scale (BES) (Gormally et al. 1982) (score > 17, which suggests binge‐eating symptoms (Marcus et al. 1985)). Severe depressive symptomatology was assessed using the Beck Depression Inventory (BDI) (Beck et al. 1961) (score < 30), plus clinical interviews using the DSM‐5 criteria. The same interviews were also used to screen participants for other enduring mental health problems, such as suicidal ideation, personality disorders, and psychosis. Participants not meeting any inclusion criteria were excluded from the trial and not randomized. Participants' BMI was checked by asking eligible participants who agreed to participate and gave their consent to send (by email) a proof of height and current weight obtained from a pharmacy scale report obtained in within the previous week. No compensation was offered to participants other than the potential benefits of the intervention on their well‐being and mental health.

### 2.3 Randomization

**FIGURE 1:**
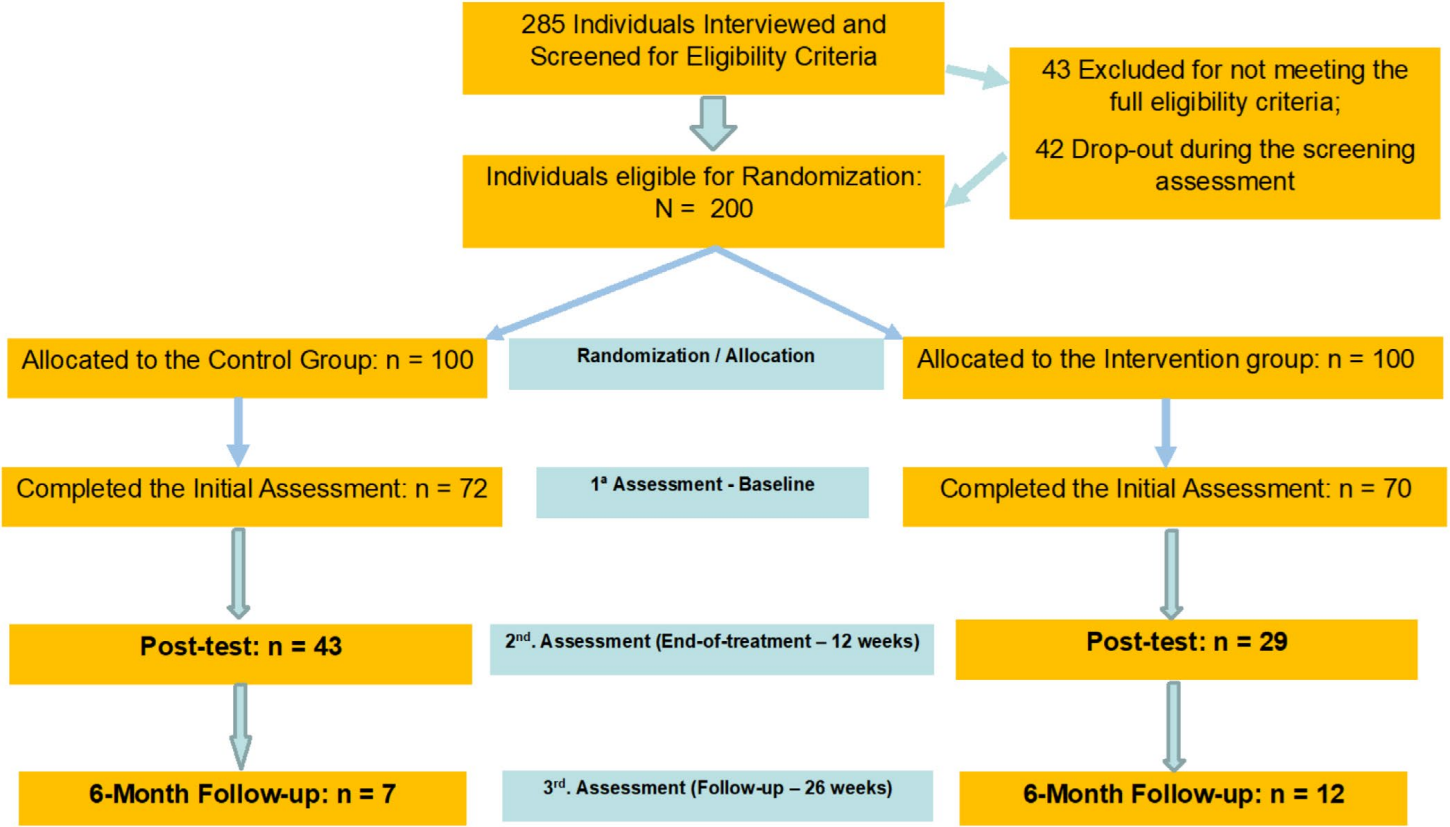
eBEfree trial flowchart.

Participants who agreed to participate in the eBEfree program were randomized into one of the two groups (eBEfree app‐based program or waiting list) in a 1:1 ratio generated through an automated computer‐based random sequence, using Microsoft Excel. The full randomization procedure was undertaken before any assessments. Full allocation concealment was not undertaken as two researchers had to be aware of which group participants had been allocated to (to manage the intervention), as well as participants who were aware of the intervention they were receiving. Two‐hundred participants were randomized, with 72 participants in the control group and 70 participants in the intervention group completing the initial assessment. The trial flowchart is presented in Figure 1.

### 2.4 Trial Arms

The current trial comprised two arms, the intervention group composed of individuals randomly assigned to start receiving the eBEfree program and the waitlist control group. The control group did not get access to the eBEfree program while the intervention group was doing the program sessions (during the program 12‐weeks). After the intervention group completed the intervention (end‐of‐treatment stage: 12‐weeks after the program has been started), the control group started the eBEfree program. The eBEfree program is a 12‐week psychological therapeutic intervention based on the BEfree program, which has been successfully tested in a previous clinical trial (Pinto‐Gouveia et al. 2019). The eBEfree program was therefore designed to cover the following key topics: (i) Mindfulness skills to promote adaptive emotion regulation and improve eating behavior regulation; (ii) Compassion skills to promote motivation that supports sustained adaptive behavior regulation; (iii) The development of psychological flexibility and the promotion of values‐based living (Acceptance and Commitment Therapy skills). The corresponding techniques for each topic were implemented sequentially in a portfolio of digital‐based tools, including a platform with multimedia resources (e.g., videos of sessions) and mobile applications to promote the engagement of users (e.g., an online chat). The entire eBEfree program was made available in a ready‐to‐be‐used app for smartphones and tablets using iOS or Android operating systems. The app was available to be installed, and afterwards, the registration was approved by the eBEfree team. The 12 sessions were designed to be done on a weekly basis, with sessions being released as the participant goes along with the program (e.g., session 2 is only available after session 1 has been completed and so on). However, all participants were able to come back to sessions already completed, to redo exercises or rewatch audios and videos. The content included in each session is presented in different formats, such as videos, animated videos, audios, and text. Videos are typically presented with eBEfree therapists (members of the research team who are trained clinical therapists) explaining concepts and positive strategies to be adopted to tackle binge‐eating behaviors and other related emotional and cognitive‐behavioral issues (e.g., emotional regulation, negative/unwanted or triggering thoughts), including mindful eating, acceptance, and self‐compassion. Animated videos have a similar purpose as the therapist videos and were designed to facilitate participants' engagement with the therapeutic activities and to present some concepts in a more ludic fashion. Audios were more focused on mindfulness and compassion‐focused therapeutic activities (e.g., meditation) to provide participants with specific guidance on how to practice mindfulness and self‐compassion in relation to themselves and, specifically, in relation to binge‐eating behaviors. Texts include therapeutic and homework activities to motivate participants to practice mindfulness and self‐compassion throughout the entire program, including in‐between sessions. The app containing the eBEfree program was therefore developed to integrate different content formats (texts, audios, videos) ready to be used by participants in the most intuitive possible way, to facilitate engagement with the program. By the middle of the program (week 6), we invited all participants who were doing the program to attend an online group session to get intermediate feedback on the program and the app and see how they were engaging with the program. Our plan was to help participants engage with the program, improve any issues reported by participants, and prevent further dropouts. The one‐off optional online group sessions were chaired by the Principal Investigator (JPG) and/or an eBEfree therapist. In addition to the online group session, we also promoted regular follow‐up email contacts with participants to check their progress with the intervention, help them with any ongoing issues while using the app, and improve their engagement with the program to prevent further dropouts. All participants were invited to provide their feedback and experience of using the eBEfree app during the program, including reporting any emotional distress experienced during the program, by emailing the eBEfree team. Finally, participants also had a Forum Chat included in the eBEfree app to share experiences with other participants anonymously. The full description of the eBEfree program and some illustrative screenshots of the eBEfree app are presented in Supporting Information.

### 2.5 Study Assessments

Study assessments for all participants were undertaken at baseline, 12 weeks after baseline assessment (end‐of‐treatment), and 26 weeks after the end‐of‐treatment point (follow‐up assessment).

#### 2.5.1 Participants Characteristics

Participant baseline demographical and clinical data including age, gender, education level, socioeconomical status, and current BMI were assessed via an online interview and an online survey. Participants' motivations for expressing interest in the eBEfree program, self‐awareness of binge‐eating behaviors, goals and expectations in relation to the program, motivation to change, and willingness to complete an online app‐based mental health program were assessed during the online individual interview by a qualified clinical psychologist part of the research team. Participant characteristics at baseline are presented in Table 1.

**TABLE 1:** Baseline characteristics.

	Total sample (*N* = 142)	Control group (*N* = 72)	Intervention group (*N* = 70)	*ρ*‐value^a^	ES (Cohen's *d*)
Age	42.5 (7.3)	42.5 (6.9)	42.7 (7.7)	0.71	0.06
Gender (female)	111 (78.1%)	59 (81.2%)	52 (74.3%)	0.37	0.07
Education level				0.16	0.16
Up to secondary school (9–12 years of education)	35 (24.6%)	(26.4%)	16 (22.9%)		
Higher education (> 12 years of education)	107 (75.4%)	53 (73.6%)	54 (77.1%)		
BMI	34.0 (5.3)	33.8 (5.3)	34.2 (5.8)	0.6	0.08
BES	24.1 (8.3)	24.4 (8.4)	23.8 (8.3)	0.7	0.06
FSCRS—self‐reassurance	17.0 (6.0)	16.7 (6.1)	17.3 (5.9)	0.5	0.1
FSCRS—feeling inadequate	19.3 (7.1)	19.6 (7.0)	19.0 (7.3)	0.7	0.07
FSCRS—self‐hating	4.7 (3.6)	4.8 (3.5)	4.6 (3.7)	0.8	0.04
SCS—self‐kindness	2.6 (0.8)	2.6 (0.8)	2.6 (0.9)	0.6	0.09
SCS—self‐judgment	3.1 (0.8)	3.1 (0.8)	3.0 (0.8)	0.3	0.2
SCS—common humanity	2.9 (0.7)	2.9 (0.8)	2.9 (0.7)	0.8	0.04
SCS—isolation	3.2 (0.9)	3.2 (0.9)	3.1 (0.9)	0.2	0.2
SCS—mindfulness	2.9 (0.7)	2.9 (0.7)	2.9 (0.7)	0.6	0.08
SCS—overidentification	3.0 (0.8)	3.1 (0.8)	3.0 (0.9)	0.6	0.09
SCS—total	2.9 (0.3)	3.0 (0.3)	2.9 (0.4)	0.2	0.2
ORWELL	96.6 (16.4)	97.0 (17.7)	96.2 (15.1)	0.8	0.05
CFQ‐BI	38.1 (16.2)	38.3 (16.8)	37.9 (15.7)	0.9	0.03
AAQ‐II	50.4 (18.4)	50.5 (18.9)	50.3 (18.0)	0.9	0.01
BDI	15.3 (8.1)	15.9 (8.7)	14.7 (7.6)	0.4	0.1
FFMQ—observing	24.5 (5.7)	23.7 (8.7)	25.4 (5.8)	0.07	0.3
FFMQ—describing	26.1 (7.2)	24.8 (7.1)	27.4 (7.1)	**0.03**	**0.4**
FFMQ—acting with awareness	24.3 (7.4)	23.7 (7.2)	24.9 (7.6)	0.3	0.2
FFMQ—non‐judgment	26.3 (5.4)	26.0 (5.4)	26.6 (5.5)	0.5	0.1
FFMQ—non‐reactivity	19.1 (3.6)	19.4 (3.5)	18.8 (3.8)	0.6	0.2
OSS	13.4 (7.6)	14.0 (7.8)	12.8 (7.3)	0.3	0.2
ELS	53.5 (9.8)	52.1 (9.8)	54.8 (9.6)	0.1	0.3

Abbreviations: AAQ‐II, acceptance and action questionnaire‐II; BES, binge‐eating scale; BMI, body mass index; CFQ‐BI, cognitive fusion questionnaire‐body image; ELS, The engaged living scale; ES, effect size; FFMQ‐15, five facet mindfulness questionnaire‐15; FSCRS, forms of self‐criticizing/attacking & self‐reassuring scale; ORWELL, obesity related well‐being‐revised; OSS, other as Shamer scale; SCS, self‐compassion scale.

^a^ *ρ*‐values from two‐tailed *t*‐tests.

#### 2.5.2 Primary Outcome

The primary outcome—binge eating symptomatology—was collected using an online standardized questionnaire, the Binge‐Eating Scale (BES) (Duarte et al. 2015; Gormally et al. 1982). The questionnaire was filled in by all participants (control and intervention group) at baseline, 12 weeks after baseline (end‐of‐treatment), and 26 weeks after the end‐of‐treatment. Full details on the BES instrument are presented in Supporting Information.

#### 2.5.3 Secondary Outcomes

Secondary outcomes were collected using online standardized questionnaires filled in by all participants (control and intervention group) at baseline, 12 weeks after baseline (end‐of‐treatment), and 26 weeks after the end‐of‐treatment. Additionally, at the end‐of‐treatment stage, all participants were invited to fill in an online survey to collect data on their experience of the eBEfree program. The main goal of the survey was to assess participants' acceptability of the eBEfree program, including preferences, perceived difficulties, quality of the contents and format, and the perceived impact on coping with difficulties associated with binge eating. Full details on secondary outcome measures are presented in Supporting Information.

##### 2.5.3.1 BMI

Participants' BMI was checked by asking participants to send (by email) a proof of height and current weight obtained from the pharmacy scale report. Due to the constraints caused by the SARS‐COV‐2 pandemic, an in‐person check of the participants' current BMI was not possible.

##### 2.5.3.2 Depression

Beck Depression Inventory (BDI) (Beck et al. 1961; Vaz Serra 1973) was used to assess depression symptomatology.

##### 2.5.3.3 Self‐Compassion

Self‐Compassion Scale (SCS) (Castilho et al. 2015a; Neff 2003) was used to assess participants' self‐compassion.

##### 2.5.3.4 Mindfulness

Five Facet Mindfulness Questionnaire‐15 (FFMQ‐15) (Baer et al. 2006; Gregório and Gouveia 2011) was used to assess mindfulness in relation to thoughts, experiences, and actions in daily life.

##### 2.5.3.5 Psychological Flexibility

The Acceptance and Action Questionnaire‐II (AAQ‐II) (Bond et al. 2011; Pinto‐Gouveia et al. 2012) was used to assess dimensions of psychological inflexibility.

##### 2.5.3.6 Wellbeing

The Obesity Related Well‐being–Revised (ORWELL‐97) (Mannucci et al. 1999; Silva et al. 2008) was used to assess participants' obesity‐related quality of Life.

##### 2.5.3.7 Self‐Criticism

Forms of Self‐Criticizing/Attacking & Self‐Reassuring Scale (FSCRS) (Castilho et al. 2015b; Gilbert et al. 2004) was used to assess participants' self‐criticism.

##### 2.5.3.8 Shame

Other as Shamer Scale (OSS) (Goss et al. 1994; Matos et al. 2015) was used to assess participants' perceptions of being negatively evaluated by others.

##### 2.5.3.9 Body Image Cognitive Fusion

Cognitive Fusion Questionnaire‐Body Image (CFQ‐BI) (Ferreira et al. 2015) was used to assess participants' cognitive fusion in relation to body image.

##### 2.5.3.10 Values‐Based Behavior

The Engaged Living Scale (ELS) (Trindade et al. 2016; Trompetter et al. 2013) was used to assess engagement with value‐driven behavior.

##### 2.5.3.11 Program Acceptability

eBEfree program acceptability was assessed using an online survey (incorporated in the online assessment protocol) to be filled out by participants at the end of the eBEfree program. Participants were also invited for an online interview with a member of our research team to provide more detailed feedback on the eBEfree program. The survey to collect participants' acceptability addressed questions on program's duration and content, the app's usability, the experience of practicing sessions' exercises, and the therapeutic impact (see Supporting Information for full details). Further details on the experience of doing each program's session was explored with the online interviews. Additionally, we have also conducted some follow‐up contacts with participants who dropped out the intervention to collect their feedback on the trial program and the app sessions. Participants' debriefing was provided in follow‐up contacts and interviews when intervention feedback was collected.

### 2.6 Sample Size Calculation

A priori sample size calculations were performed with G‐Power (version 3.1.9.6) for a mixed‐design ANOVA with one interaction: between group (2 groups: control vs. treatment) vs. assessments (3 repeated measurements: baseline; end‐of‐treatment; 26 weeks follow‐up). Considering the lack of prior evidence on the intervention's effectiveness, the mixed‐design ANOVA was set for a medium effect size (Cohen's *F* = 0.25), a statistical Power (1 − *β*) of 0.95, an α of 0.5, an intra‐correlation coefficient of 0.5, and nonsphericity correction (ɛ) of 1, resulting in a minimum total sample size of 44 subjects, and a critical *F* = 3.11. Assuming a 20%–30% drop‐out rate, from previous literature (Linardon et al. 2020) and our previous trial (Pinto‐Gouveia et al. 2017), the goal for recruitment was a sample size of 70 participants.

### 2.7 Statistical Analysis

All statistical analyzes were undertaken with R (version 4.3.1; RStudio 2023.12.0), following intention‐to‐treat principles by including participants in the condition they were randomized to at baseline. Attrition rates between groups were compared using the McNemar χ^2^ test. Mixed‐design ANOVA models were undertaken to examine the main interaction effect (Group*Assessments) for the outcome measures only for per‐protocol analysis (the analysis to compare groups that includes only those individuals who completed the treatment (or control condition) originally allocated), using the *ez* R package. Due to the high attrition rate at 26 weeks follow‐up, per‐protocol analysis was only conducted for two assessment points (baseline + end‐of‐treatment). As ANOVA models do not account for fixed and random effects within the same model, the need and relevance for running linear mixed models was inspected by running two separate models using maximum‐likelihood estimation: an intercept‐only generalized least squares model and a random‐intercept‐only model. Models were then compared for the best Akaike Information Criterion (AIC) value, with the random‐intercept‐only model showing a lower AIC value and a *ρ*‐value < 0.001, i.e., suggesting a better fit for a linear mixed model. Linear mixed models were, therefore, adopted for all intention‐to‐treat (ITT) analysis, using the *lme4* (Bates et al. 2015a) and *lme* (Pinheiro et al. 2020) R packages.

Three different linear mixed models with three covariates, age, gender, and education, were investigated for primary outcomes and then compared for the best AIC value using a likelihood ratio test: (1) a random‐intercept model; (2) a random intercept plus random slopes at the individual level; and (3) a random intercept plus random slopes at the group level. Results suggested no significant differences between models (*ρ* > 0.05) so the simplest model (random‐intercept—see Equation (1)) was then chosen as the final model, as it entails less complexity and is less likely to get convergence issues, particularly in studies with modest sample sizes (Bates et al. 2015b; West et al. 2022). ITT analysis assuming data missing at random was, therefore, undertaken using random‐intercept models for primary and secondary outcomes. Statistical assumptions for linear mixed models were inspected, with results suggesting models were reliable, including normal distribution and equal variance of conditional residuals, normal distribution of random effects, and model convergence.

Equation (1) Random‐Intercept Model

(1) $$\gamma_{\mathrm{ij}}=\beta_{00}+\beta_{1}X1_{\mathrm{ij}}+\beta_{2}X2_{\mathrm{ij}}+\beta_{3}X3_{\mathrm{ij}}+\beta_{4}X4_{\mathrm{ij}}+\beta_{5}X2_{\mathrm{ij}}X3_{\mathrm{it}}+U_{0i}+{Ɛ}_{\mathrm{ti}}$$

With *Y* being the outcome measure, and *i* = 1, …, *n* individuals, *j* = 1–2 groups, *t* = 1–3 temporal assessments (baseline + end‐of‐treatment + 26‐weeks follow‐up). Fixed effects are expressed as β (assessment (*X*1), group (*X*2), age (*X*3), sex (*X*4), and interaction assessment*group (*X*1*X2)). *U*_0*j*_ represents the random effects at the individual level (intercept), and *Ɛ*_*ti*_ the residuals.

Sensitivity analysis for the primary outcome (Binge Eating Scale) was performed using Bayesian hierarchical (random‐intercept) models (see Equation (2)). Sensitivity analysis was only performed for the primary outcome (BES scores) because it is the main outcome for assessing treatment efficacy in our study, and because to the best of our knowledge this is the only outcome (included in our study) for which we have available evidence on the potential magnitude of treatment effect associated with similar digital mental health interventions for binge‐eating. That prior evidence is crucial for Bayesian estimation as the uncertainty is modeled directly and by integrating prior knowledge on the parameters of interest (Congdon 2019; Gelman et al. 2013). Bayesian statistics have become popular in medical research and clinical trials (Ferreira et al. 2022; Lammers et al. 2023; Seixas et al. 2014), and are regarded as a powerful statistical method for handling relatively small samples entailing clustered data at higher precision, in comparison with the frequentist statistical approach (Congdon 2019; Gelman et al. 2013). The sensitivity analysis included Bayesian models using different weakly informative priors, plus more informative priors for the coefficients of interest (see Equation (2)). Noninformative priors were not computed, as recent literature has highlighted the potential overestimation of the magnitude of effects resulting from the use of noninformative flat priors, which contradicts the “non‐informative” assumption inherent to these priors (Gelman et al. 2017; Gelman and Carlin 2014; Lemoine 2019; van Zwet and Gelman 2022). Informative prior distributions were chosen according to the available literature. The prior distribution of the coefficient for the intercept (β_0_ ~ *N*(22.3, 6.0)) considered the mean and SD for BES in previous validation studies with adults with binge‐eating and obesity (Gormally et al. 1982; Timmerman 1999). Two different prior distributions of the coefficient for the treatment effect (X2_ijt_ X3_ijt_β_5_) were set. One was according to a meta‐analysis of e‐mental health intervention studies for binge‐eating which suggested an effect size of 0.29 (Linardon et al. 2020) (~*N*(−0.15, 5.5)), and a more recent large randomized controlled trial suggesting an effect size of 0.60 (~*N*(−0.29, 17.0)) (Linardon et al. 2023).

All Bayesian models were run with the *brms* R package which uses the Stan probabilistic programming language and implements the No‐U‐Turn Sampler (NUTS) extension of the Hamiltonian Monte Carlo algorithm (Bürkner 2017, 2018). Models were set with 10,000 iterations, 5000 warm‐up, 4 chains, 1 core and 1 thins. As Bayesian models with *brms* cannot handle missing data, multiple imputation was performed using fully conditional specification implemented through the MICE algorithm which adopts multivariate imputation by chained equations, using the *mice* R package (van Buuren and Groothuis‐Oudshoorn 2011). 50 imputations were used for all outcome measures with missing data. Data were imputed only for end‐of‐treatment outcome missing data, as there were no missing data at baseline for the outcome, and also no missing data in any covariates. Multiple imputation for missing data at 26 weeks follow‐up was not performed to avoid a biased analysis, as the attrition rate from baseline to follow‐up was greater than 85%.

Equation (2) Bayesian Multilevel Model

(2) $$\gamma_{\mathrm{ijt}}=X_{\mathrm{ijt}}\beta_{0\ldots 4}+Z_{\mathrm{ijt}}b_{i}+{Ɛ}_{\mathrm{ij}}$$

Weakly‐informative Priors: (1) β ~ *N*(0, 10), *b*_*ij*_ ~ Cauchy(0, 10); (2) *β* ~ *N*(0, 5), *b*_*ij*_ ~ Cauchy(0, 5). Informative Priors: (1) *β*_0_ ~ *N*(22.3, 6), *X*2_*ijt*_ *X*3_*ijt*_ *β*_5_ ~ *N*(−0.15, 5.5), *b*_*ij*_ ~ Cauchy(0, 5); (2) *β*_0_ ~ *N*(22.3, 6), *X*2_ijt_ *X*3_ijt_ *β*_5_ ~ *N*(−0.29, 17), *b*_*ij*_ ~ Cauchy(0, 5).

With ***γ*** representing the outcome for individuals (*i*), groups (*j*) and assessments (*t*), $X_{\mathrm{ijt}}\beta_{0\ldots 4}$ representing the coefficients for all fixes effects as stated in Equation (1), $Z_{\mathrm{ijt}}b_{i}$ the random effects at individual level, and ${Ɛ}_{\mathrm{ij}}$ the residuals. Coefficients' prior distributions set for ~ (mean, SD).

## 3 Results

### 3.1 Baseline Characteristics

The sample baseline characteristics are presented in Table 1. Most of our sample was composed of female individuals, with a high level of education (> 12 years of education), an elevated body mass index (within the obesity range) and high binge‐eating symptomatology (according to the Binge Eating Scale). No significant differences between control and intervention groups were found for the primary and secondary outcome measures (*p* > 0.05) except for the FFMQ dimension of mindfulness *Describing*, in which the intervention group showed slightly higher scores (Table 1).

### 3.2 Study Attrition

One hundred forty‐two individuals (72 in the control group and 70 in the intervention group) completed all baseline assessments, from which 72 individuals (43 in control group and 29 in the intervention group) completed all assessments at the end‐of‐treatment (post‐test) stage. The 29 participants in the intervention group who completed the end‐of‐treatment assessment had completed the 12‐week eBEfree program. At the 26‐week follow‐up, 19 individuals completed all assessments (7 in the control group and 12 in the intervention group). The attrition rates were, therefore, 50% at the end‐of‐treatment stage (in relation to baseline: 58.5% in the intervention group; 40.3% in the control group), and 74% at the 26‐week follow‐up (in relation to end‐of‐treatment: 58.6% in the intervention group; 83.7% in the control group). McNemar test suggested significant differences between groups for the attrition rate, with greater attrition in the treatment group at the end‐of‐treatment stage (McNemar *χ* ^2^ = 5.98, *ρ* = 0.01), and greater attrition in the control group at the 26‐week follow‐up (McNemar *χ* ^2^ = 12.25, *ρ* < 0.001). *t*‐tests showed no significant differences (*ρ* > 0.05) for all primary and secondary outcomes at baseline between individuals who completed all end‐of‐treatment assessments and individuals who dropped out of the trial. No statistical differences were found for demographics between participants who completed the program and those who dropped out.

### 3.3 Post‐Treatment and Follow‐Up Efficacy

#### 3.3.1 Primary Outcome

**FIGURE 2:**
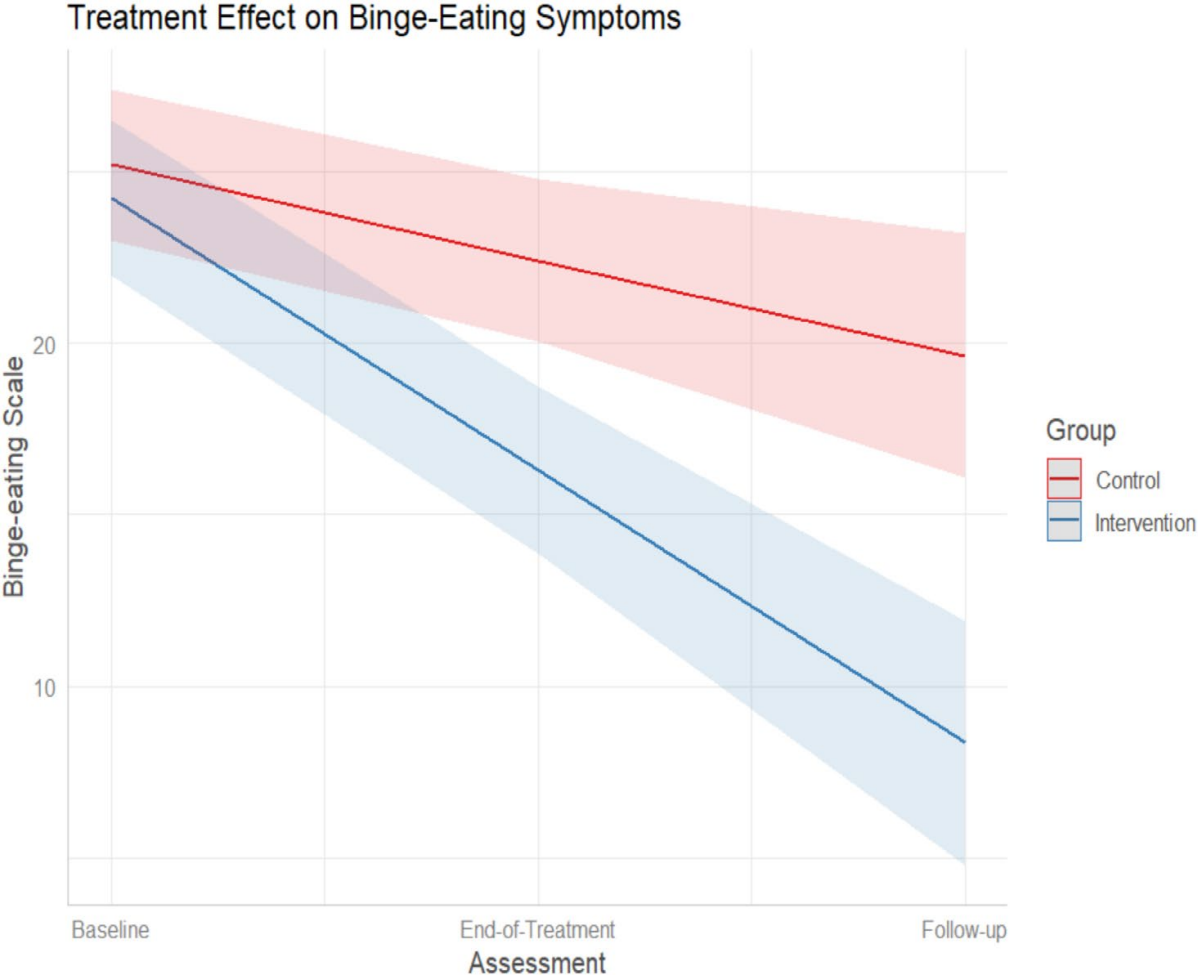
eBEfree treatment effect on symptoms of binge‐eating (results from linear mixed models).

ITT analysis for the primary outcome (BES) using a random intercept model showed a significant reduction in binge eating symptomatology from baseline to end‐of‐treatment, and also from end‐of‐treatment to 26‐weeks follow‐up (Table 2; Figure 2). The effect sizes were large at end‐of‐treatment (*g* = −1.17) and at follow‐up (*g* = −1.08).

**TABLE 2:** Intention‐to‐treat analysis.

	Control group		Intervention group		Effect size (Hedge's g)	β (95% CI) treatment effect^a^	SE^a^	*T*‐statistic^a^	*ρ*‐value^a^
	*N*	*M* (SD)	*N*	*M* (SD)					
BES									
Baseline	72	24.4 (8.4)	70	23.8 (8.3)					
End‐of‐treatment	43	22.6 (8.4)	29	12.3 (8.3)	−1.17	−9.49 (−12.64 to −6.35)	1.62	−5.84	**< 0.0001**
6‐Month follow‐up	7	20.4 (14.4)	12	12.4 (8.7)	−1.08	−6.01 (−11.69 to −0.36)	2.92	−2.06	**0.042**
BMI									
Baseline	72	33.8 (5.3)	70	34.2 (5.8)					
End‐of‐treatment	43	32.8 (4.8)	29	31.7 (4.8)	‐ 0.24	−1.15 (−2.25 to −0.05)	0.57	−2.03	**0.045**
6‐Month follow‐up	7	31.8 (6.1)	12	30.5 (4.6)	−0.28	−1.96 (−3.92 to −0.01)	1.01	−1.93	0.057
FSCRS—self‐reassurance									
Baseline	72	16.7 (6.1)	70	17.3 (5.9)					
End‐of‐treatment	43	16.2 (6.3)	29	18.9 (6.2)	0.43	1.73 (−0.45 to 3.92)	1.13	1.53	0.129
6‐Month follow‐up	7	16.1 (3.8)	12	20.3 (5.3)	0.52	1.94 (−1.98 to 5.90)	2.03	0.96	0.340
FSCRS—feeling inadequate									
Baseline	72	19.6 (7.0)	70	19.0 (7.3)					
End‐of‐treatment	43	19.3 (6.5)	29	15.2 (8.1)	−0.56	−4.10 (−6.49 to −1.70)	1.23	−3.31	**0.001**
6‐Month follow‐up	7	22.0 (7.7)	12	15.5 (7.2)	−0.61	−0.70 (−5.06 to 3.59)	2.22	−0.32	0.751
FSCRS—self‐hating									
Baseline	72	4.8 (3.5)	70	4.6 (3.7)					
End‐of‐treatment	43	5.4 (4.1)	29	3.4 (2.9)	−0.57	−1.89 (−3.03 to −0.75)	0.59	−3.21	**0.002**
6‐Month follow‐up	7	6.1 (2.9)	12	3.2 (2.8)	−0.63	−0.10 (−2.18 to 1.95)	1.06	−0.10	0.925
SCS—self‐kindness									
Baseline	72	2.6 (0.8)	70	2.6 (0.9)					
End‐of‐treatment	43	2.6 (0.8)	29	2.9 (0.8)	0.35	0.23 (−0.04 to 0.42)	0.14	1.64	0.104
6‐Month follow‐up	7	2.7 (0.6)	12	2.9 (1.1)	0.33	−0.06 (−0.54 to 0.42)	0.25	−0.24	0.808
SCS—Self‐judgment									
Baseline	72	3.1 (0.8)	70	3.0 (0.8)					
End‐of‐treatment	43	3.1 (0.7)	29	2.9 (1.0)	−0.32	−0.21 (−0.46 to 0.04)	0.13	−1.60	0.116
6‐Month follow‐up	7	3.4 (0.4)	12	2.5 (1.1)	−0.48	0.0009 (−0.46 to 0.45)	0.24	0.004	0.997
SCS—Common humanity									
Baseline	72	2.9 (0.8)	70	2.9 (0.7)					
End‐of‐treatment	43	2.8 (0.7)	29	3.0 (0.6)	0.27	0.28 (−0.01 to 0.56)	0.15	1.89	0.063
6‐Month follow‐up	7	3.3 (0.6)	12	2.8 (0.9)	0.11	−0.48 (−0.99 to 0.03)	0.26	−1.82	0.072
SCS—isolation									
Baseline	72	3.2 (0.9)	70	3.1 (0.9)					
End‐of‐treatment	43	2.8 (0.7)	29	3.0 (0.6)	0.42	0.04 (−0.29 to 0.36)	0.17	0.23	0.819
6‐Month follow‐up	7	4.0 (1.0)	12	2.7 (1.1)	−0.57	−0.06 (−0.66 to 0.51)	0.30	−0.22	0.826
SCS—Mindfulness									
Baseline	72	2.9 (0.7)	70	2.9 (0.7)					
End‐of‐treatment	43	2.8 (0.7)	29	3.1 (0.7)	0.47	0.28 (−0.00 to 0.55)	0.14	1.98	0.050
6‐Month follow‐up	7	3.0 (0.8)	12	3.1 (0.7)	0.40	0.06 (−0.42 to 0.55)	0.25	0.25	0.081
SCS—Overidentification									
Baseline	72	3.1 (0.8)	70	3.0 (0.9)					
End‐of‐treatment	43	3.1 (0.8)	29	2.8 (1.0)	−0.30	−0.22 (−0.50 to 0.06)	0.14	−1.50	0.136
6‐Month follow‐up	7	3.3 (0.6)	12	2.5 (0.8)	−0.46	−0.04 (−0.55 to 0.46)	0.26	−0.15	0.879
SCS—TOTAL									
Baseline	72	3.0 (0.3)	70	2.9 (0.4)					
End‐of‐treatment	43	2.9 (0.3)	29	2.9 (0.3)	0.07	0.06 (−0.07 to 0.20)	0.07	0.95	0.346
6‐Month follow‐up	7	3.3 (0.4)	12	2.8 (0.3)	−0.35	−0.14 (−0.38 to 0.10)	0.12	−1.13	0.263
ORWELL									
Baseline	72	97.0 (17.7)	70	96.2 (15.1)					
End‐of‐treatment	43	96.6 (16.0)	29	89.4 (15.1)	−0.46	−4.55 (−9.48 to 0.34)	2.53	−1.80	0.075
6‐Month follow‐up	7	99.3 (15.5)	12	86.3 (20.4)	−0.52	−2.26 (−11.14 to 6.53)	4.55	−0.50	0.621
CFQ‐BI									
Baseline	72	38.3 (16.8)	70	37.9 (15.7)					
End‐of‐treatment	43	37.9 (13.3)	29	32.9 (13.8)	−0.36	−4.42 (−9.14 to 0.30)	2.44	−1.81	0.073
6‐Month follow‐up	7	38.0 (16.5)	12	29.8 (16.2)	−0.41	−2.00 (−10.51 to 6.46)	4.38	−0.46	0.649
AAQ‐II									
Baseline	72	50.5 (18.9)	70	50.3 (18.0)					
End‐of‐treatment	43	46.5 (15.3)	29	41.5 (16.9)	−0.31	−4.97 (−10.66 to 0.72)	2.94	−1.69	0.094
6‐Month follow‐up	7	47.9 (19.0)	12	40.2 (20.9)	−0.33	−2.65 (−12.91 to 7.55)	5.27	−0.50	0.616
BDI									
Baseline	72	15.9 (8.7)	70	14.7 (7.6)					
End‐of‐treatment	43	17.2 (8.9)	29	11.5 (7.9)	−0.67	−3.91 (−7.76 to −0.21)	1.89	−2.07	**0.040**
6‐Month follow‐up	7	17.4 (9.1)	12	10.2 (8.2)	−0.71	−2.74 (−9.42 to 3.83)	3.39	−0.81	0.420
FFMQ—observing									
Baseline	72	23.7 (8.7)	70	25.4 (5.8)					
End‐of‐treatment	43	23.2 (5.7)	29	26.7 (5.9)	0.58	1.54 (−1.29 to 4.37)	1.46	0.70	0.294
6‐Month follow‐up	7	24.0 (6.8)	12	27.3 (4.7)	0.61	1.85 (−3.23 to 6.91)	2.61	0.71	0.480
FFMQ—describing									
Baseline	72	24.8 (7.1)	70	27.4 (7.1)					
End‐of‐treatment	43	25.4 (7.0)	29	29.1 (7.7)	0.49	0.64 (−2.61 to 3.92)	1.69	0.38	0.702
6‐Month follow‐up	7	23.4 (7.3)	12	28.8 (8.0)	0.52	0.78 (−5.06 to 6.69)	3.02	2.58	0.796
FFMQ—acting with awareness									
Baseline	72	23.7 (7.2)	70	24.9 (7.6)					
End‐of‐treatment	43	24.3 (6.9)	29	26.1 (8.4)	0.24	0.40 (−2.91 to 3.72)	1.71	0.23	0.817
6‐Month follow‐up	7	24.0 (5.8)	12	25.0 (7.8)	0.21	−0.76 (−6.71 to 5.20)	3.07	−0.25	0.804
FFMQ—non‐judgment									
Baseline	72	26.0 (5.4)	70	26.6 (5.5)					
End‐of‐treatment	43	26.0 (5.3)	29	27.3 (6.2)	0.21	0.60 (−1.95 to 3.15)	1.31	0.46	0.649
6‐Month follow‐up	7	21.4 (3.8)	12	26.7 (6.1)	0.30	2.65 (−1.91 to 7.29)	2.36	1.12	0.262
FFMQ—non‐reactivity									
Baseline	72	19.4 (3.5)	70	18.8 (3.8)					
End‐of‐treatment	43	18.3 (3.8)	29	20.0 (5.0)	0.38	2.31 (0.51 to 4.10)	0.93	2.49	**0.014**
6‐Month follow‐up	7	20.9 (4.1)	12	20.1 (2.9)	0.33	0.07 (−3.15 to 3.29)	1.66	0.04	0.965
OSS									
Baseline	72	14.0 (7.8)	70	12.8 (7.3)					
End‐of‐treatment	43	13.7 (7.1)	29	10.3 (6.2)	−0.51	−1.60 (−4.93 to 2.15)	1.70	−0.94	0.349
6‐Month Follow‐up	7	16.9 (6.5)	12	7.8 (5.5)	−0.69	−3.81 (−9.94 to 2.15)	3.06	−1.24	0.216
ELS									
Baseline	72	52.1 (9.8)	70	54.8 (9.6)					
End‐of‐treatment	43	51.5 (8.4)	29	59.4 (10.0)	0.84	4.81 (−0.07 to 9.60)	2.45	1.96	0.051
6‐Month follow‐up	7	53.4 (10.1)	12	62.0 (9.3)	0.91	4.73 (−3.74 to 13.30)	4.38	1.08	0.282

Abbreviations: AAQ‐II, acceptance and action questionnaire‐II; BES, binge‐eating scale; BMI, body mass index; CFQ‐BI, cognitive fusion questionnaire‐body image; ELS, The engaged living scale; FFMQ‐15, five facet mindfulness questionnaire‐15; FSCRS, forms of self‐criticizing/attacking & self‐reassuring scale; ORWELL, obesity related well‐being‐revised; OSS, other as Shamer scale; SCS, self‐compassion scale.

^a^ Parameters' values from linear mixed models (random‐intercept), adjusted for sex and education level.

#### 3.3.2 Secondary Outcomes

**FIGURE 3:**
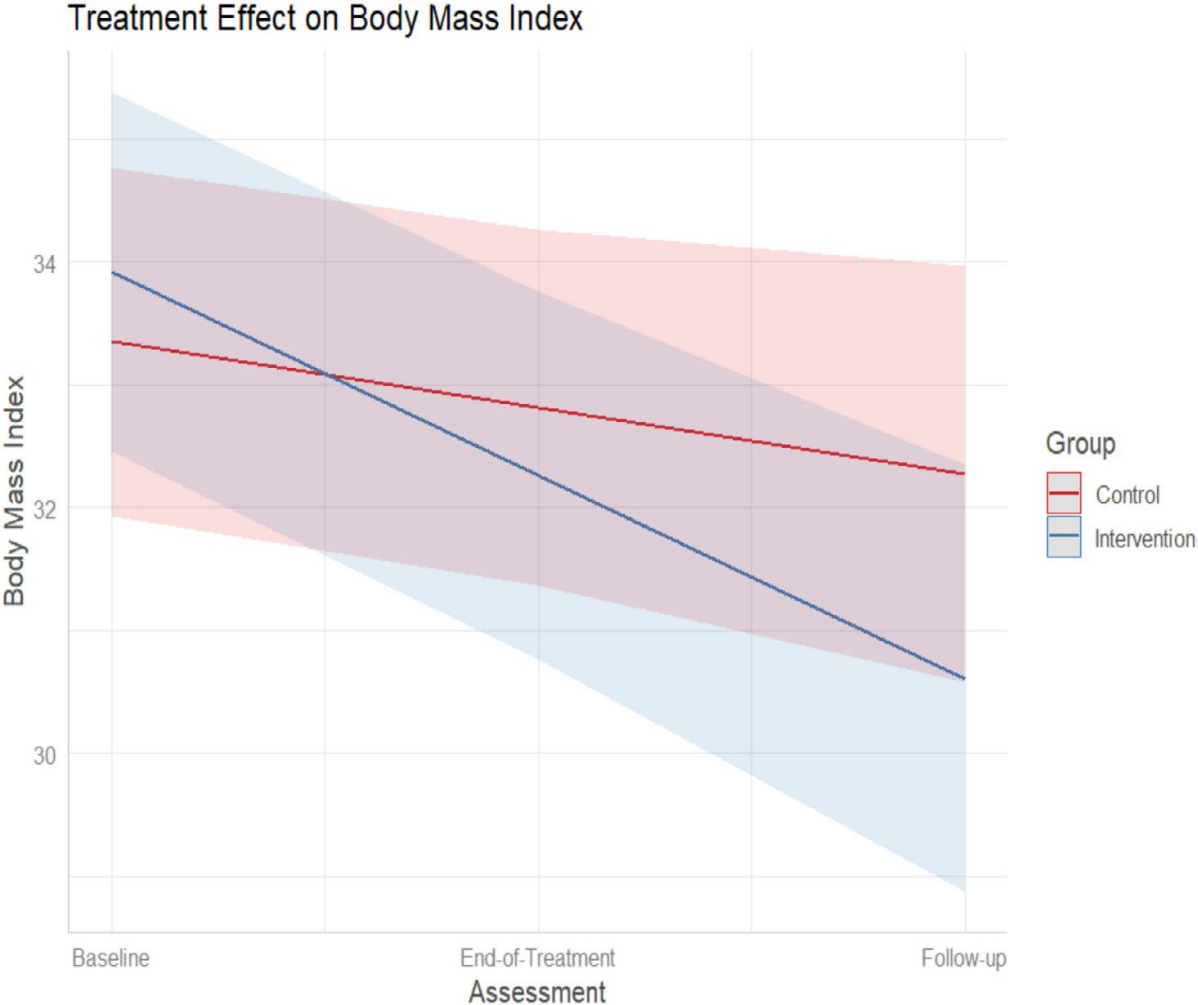
eBEfree treatment effect on body mass index (results from linear mixed models).

**FIGURE 4:**
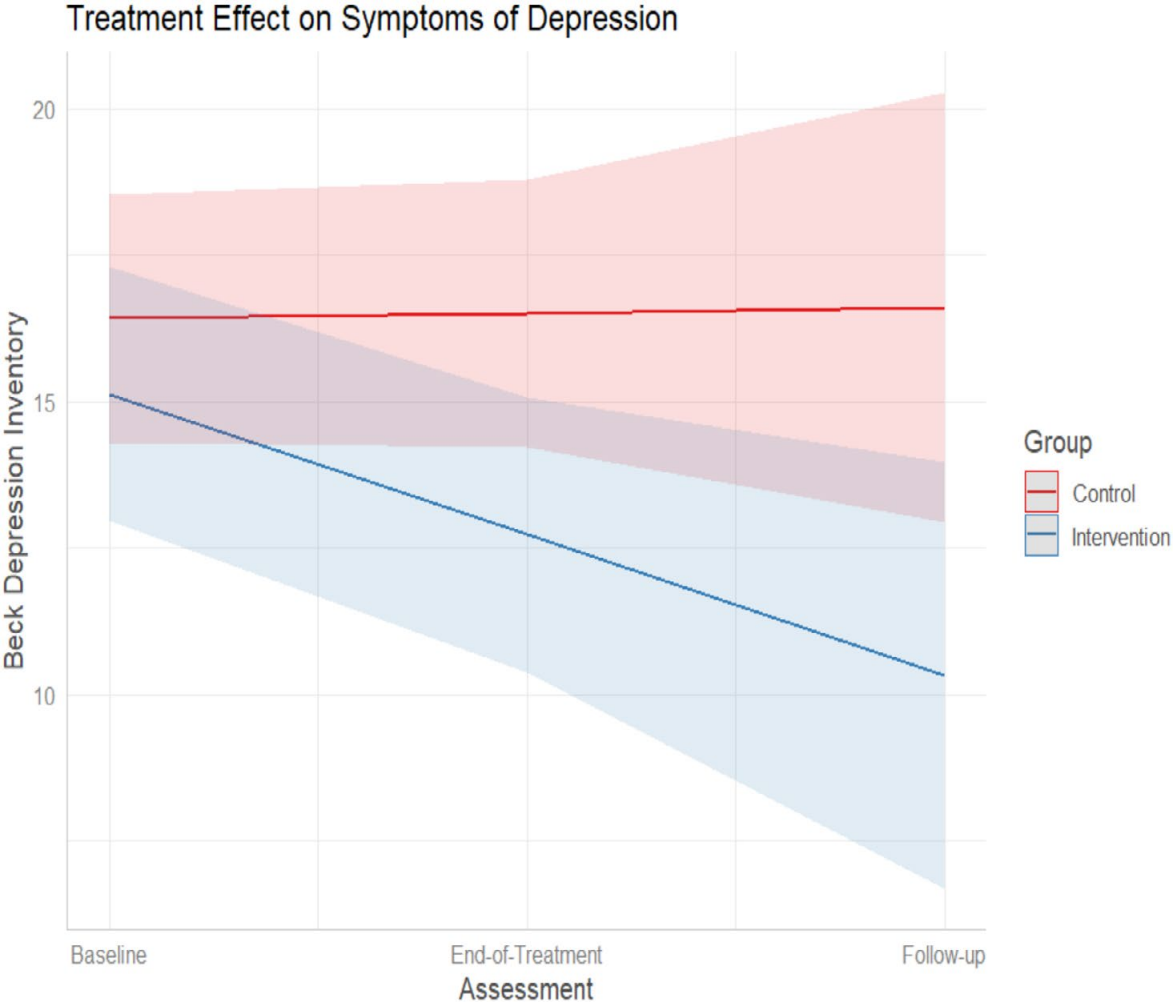
eBEfree treatment on symptoms of depression (results from linear mixed models).

ITT analyzes for all secondary outcomes are presented in Table 2. At end‐of‐treatment, the intervention was associated with a significantly lower BMI (Figure 3), significantly lower scores on the FSCRS dimensions of self‐criticism, inadequate self, and hated self, significantly lower depression symptomatology (BDI) (Figure 4), and significantly greater scores on the FFMQ dimension of mindfulness *Non‐reactivity*, with effect sizes (Hedge's *g*) ranging from 0.24 to 0.67. At 26‐weeks follow‐up, the scores in the outcomes did not significantly change in relation to the end‐of‐treatment point, suggesting that the benefits of the intervention were maintained during the 26‐weeks follow‐up. The effect sizes (*g*) for secondary measures at end‐of‐treatment and at 26‐weeks follow‐up ranged from low (BMI; FFMQ) to medium (BDI, FSCRS). No significant effects were detected for the remaining secondary measures, including the other dimensions of mindfulness (FFMQ) and self‐criticism (FSC), and for all dimensions of self‐compassion (SCS), psychological flexibility (AAQ‐II), wellbeing (ORWELL‐97), Shame (OSS), cognitive fusion (CFQ‐BI), and values‐based behavior (ELS).

### 3.4 Sensitivity Analysis

The Bayesian analysis for the primary outcome is presented in Table 3. Results confirmed the treatment effect for reducing binge‐eating symptomatology, lowering BES scores at end‐of‐treatment (credible intervals not crossing zero for the interaction group*assessment), although suggesting a slightly smaller coefficient for the treatment effect in comparison with the frequentist multilevel model (−5.07 vs. −9.49). All models converged, with Rhat values equal to 1. Additional data on Bayesian model diagnostics is presented in Supporting Information. The model diagnostics for both studies are presented in the Supporting Information, including the posterior predictive density of model parameters suggesting normality of the posterior distribution, and trace plots and the posterior predictive checks plot suggesting that the model converged.

**TABLE 3:** Bayesian multilevel models for the primary outcomes (Binge‐eating symptomatology).

Predictors	Model with weakly‐informative priors ~*N*(0, 10)			Model with weakly‐informative priors ~*N*(0, 5)			Model with informative priors (a)			Model with informative priors (b)		
	*β*	SD	CrI (95%)	*β*	SD	CrI (95%)	*β*	SD	CrI (95%)	*β*	SD	CrI (95%)
Group [intervention]	−0.34	1.42	−3.13 to 2.45	−0.40	1.37	−3.09 to 2.29	−0.38	1.37	−3.07 to 2.31	−0.26	1.38	−2.96 to 2.44
Assessment [end‐of‐treatment]	−3.61	1.15	−5.90 to −1.38	−3.63	1.11	−5.85 to −1.48	−3.60	1.11	−5.82 to −1.44	−3.49	1.13	−5.73 to −1.30
Sex [male]	−2.78	1.65	−6.00 to 0.45	−2.60	1.58	−5.68 to 0.51	−2.60	1.58	−5.69 to 0.52	−2.60	1.58	−5.68 to 0.51
Education [higher education]	0.32	1.60	−3.48 to 2.80	0.31	1.54	−3.34 to 2.70	0.31	1.54	−3.34 to 2.69	0.31	1.54	−3.34 to 2.69
Assessment [end‐of‐treatment]: group [intervention]	−5.15	1.58	−8.28 to −2.11	−5.00	1.51	−7.99 to −2.08	−5.07	1.52	−8.07 to −2.13	−5.30	1.57	−8.41 to −2.28
Random effects												
τ_00 subjid_	6.50 (0.70); CrI: [4.99–7.80]			6.48 (0.70); CrI: [4.96–7.77]			6.47 (0.70); CrI: [4.95–7.76]			6.47 (0.70); CrI: [4.95–7.76]		
Sigma (residual SD)	5.47 (0.51); CrI: [4.63–6.69]			5.46 (0.51); CrI: [4.63–6.68]			5.46 (0.51); CrI: [4.63–6.68]			5.46 (0.51); CrI: [4.63–6.68]		
Observations	284			284			284			284		

*Note*: Parameters are summarized using the mean (*β*) and the standard deviation (SD) of the posterior distribution, with the corresponding 95% credible intervals. (a) Model with *X*2_*ijt*_ *X*3_*ijt*_ *β*_5_ ~ *N*(−0.15, 5.5), (b) Model with *X*2_*ijt*_ *X*3_*ijt*_ *β*_5_ ~ *N*(−0.29, 17).

### 3.5 Per‐Protocol Analysis

Mixed ANOVA models with only complete assessments confirmed the ITT results from linear mixed models, with the following exceptions: significant interactions between assessments (baseline × end‐of‐treatment) and group (control × treatment), in favor of the treatment group at end‐of‐treatment, with greater scores on the SCS dimension “common humanity” (*F*(1,70) = 4.10, *ρ* = 0.047); and a non‐significant treatment interaction for BDI scores (symptoms of depression) (*F*(1,70) = 2.14, *ρ* = 0.15). Full per‐protocol results are presented in Supporting Information.

### 3.6 Intervention Usage and Acceptability

Among participants who had been assigned to receive the intervention and had completed the baseline assessment (*N* = 70), 67 (95.7%) downloaded the app and logged in at least once. Of those who accessed and started the eBEfree program, 55 (82%) completed at least 25% of the program (3 sessions), 37 (55%) completed 50% of the program (6 sessions), 34 (51%) completed 75% of the program (9 sessions), and 29 (43%) completed the entire program (12 sessions in 12 weeks). The mean number of sessions completed among all participants was 5.5 (SD = 2.4).

The full report on participants' acceptability for all participants who completed the eBEfree program is presented in Supporting Information. In general, most participants found the program's format and length adequate, easy to use, and helpful. More than 80% of participants rated the quality of the program's sessions as positive and helpful. For the majority of participants, the preferred program's content format was audios, followed by animated videos and videos with therapists. Most participants (59%) said they were practicing session exercises once a week. Most participants (> 70%) experienced improvements in the way they were coping with their difficulties, including being less self‐critical, greater self‐acceptance, living more in the present, being more self‐compassionate, and better coping with emotions.

Regarding participants who dropped out of the trial intervention, 10 participants gave their feedback about their experiences of doing the eBEfree program. Main reasons pointed out for having given up the program included: lack of human contact; the app could be more interactive; technical issues that sometimes limited access to the contents; greater self‐awareness of binge‐eating episodes which made them feel more guilty and therefore avoidant in relation to the eBEfree sessions; and lack of motivation because the therapeutic gains were not seen in the short term. On the positive side, these participants highlighted: the freedom to do the program session whenever they feel like it; greater self‐awareness of emotional and behavioral issues triggering binge‐eating; the sessions' video and audio contents, including mindfulness exercises, which helped them develop new strategies to deal with binge‐eating behaviors; and the intermediate group sessions.

## 4 Discussion

The current study examined the effectiveness of a digital version of the BEfree program (Pinto‐Gouveia et al. 2017, 2019) the eBEfree—an app‐based psychological intervention for binge eating symptoms in individuals with overweight and obesity, which integrates components of psychoeducation, mindfulness, compassion, and values‐based actions derived from the ACT approach (Hayes et al. 1999).

In a remote parallel‐group randomized trial, participants in the intervention group were given access to the 12‐session weekly digital intervention; participants in the waitlist control group were assigned to be in a waiting list. Effects of the intervention were examined at post‐intervention (12 weeks) and at 26 weeks follow‐up. ITT findings revealed that at post‐treatment participants in the eBEfree presented a significant reduction in binge eating symptomatology (primary outcome of the study). This reduction was also shown at 26‐weeks follow up. These results had large effect sizes. In terms of the secondary outcomes, results also revealed that the intervention group presented significant reductions in BMI, depressive symptomatology, and self‐criticism in the forms inadequate self and hated self. There were also significant increases in the mindfulness facet non‐reactivity. This may be interpreted as a result from the specific content of the eBEfree intervention, which frames its mindfulness exercises as tools for developing noticing and non‐reactivity skills, in order to choose valued action, which might then reflect in the significant result of the non‐reactivity facet. It is also worth noting that concerns have been raised regarding the validity of some facets, namely according to previous experience with meditation (Christopher et al. 2012). Given that we used the shorter version of the FFMQ, these results should be interpreted with even more caution due to smaller reliability than the long version, and potential ceiling effects (Pelham III et al. 2019). These results were maintained at the 26‐weeks follow up assessment. Per‐protocol analysis confirmed the ITT findings, except for a significant effect for the SCS dimension common humanity, and the effect on depressive symptoms was non‐significant. Moreover, results indicated an overall trend herein participants enrolled in the eBEfree intervention showed increases in self‐compassion, self‐reassurance, mindfulness, and values‐based behavior; and decreases in psychological inflexibility, shame, and in the impact of overweight/obesity in quality of life.

Overall, these results are similar to the findings of the original face‐to‐face BEfree program (Pinto‐Gouveia et al. 2017, 2019) and suggest the efficacy of this integrated psychological approach in reducing binge eating symptomatology. In comparison with the BEfree program, at end‐of‐treatment, the eBEfree intervention showed an effect size a bit lower for reduction of binge‐eating symptoms (1.17 vs. 1.95) and for lowering BMI (0.24 vs. 0.52). However, regarding binge‐eating symptomatology, both effect sizes are high and may represent a positive clinically significant change toward a healthy and better‐adjusted eating behavior. Furthermore, considering the cost and resource intensiveness of face‐to‐face intervention, the eBEfree program offers an attractive alternative or complementary low‐intensity resource to traditional forms of intervention. The eBEfree intervention seems to be efficacious in reducing binge‐eating symptoms which are known to be potentially triggered and maintained by factors such as negative affect (Dingemans et al. 2017; Giel et al. 2022; Leehr et al. 2015) and self‐criticism (Duarte et al. 2014; Williams and Levinson 2022). Moreover, the eBEfree intervention seems to promote participants' mindfulness capacity to remain non‐reactive to internal experiences, allowing these experiences to arise and flow as they are without getting caught up in them. Research has found mindfulness and in particular this non‐reactivity trait to be a potential protective mechanism for binge eating and a mediator of treatment outcome (Kristeller 2013; Sala and Levinson 2017).

Overall, the psychometric measurements' findings were corroborated by the acceptability survey that participants completed at the end of the intervention. Most participants reported that the eBEfree was useful, and that the way they coped with their difficulties improved. Specifically, they reported that the intervention helped them being less self‐critical, more accepting of their inner experience and being better able to cope with negative emotions, living more in the present moment, and being more self‐compassionate. Most participants also found the program's format and length to be adequate, and the app to be easy to use.

As governments currently emphasize the need for citizens to be proactive in the prevention and treatment of preventable diseases, the development and availability of accessible interventions becomes a public health priority (World Health Organization and United Nations Children's Fund (UNICEF) 2018; World Health Organization 2023). Moreover, the surge in eating disorders post COVID‐19, and in particular binge eating (Caldiroli et al. 2023), stresses the need for evidence‐based timely healthcare options to the wider community struggling with binge eating. The current study findings' support the effectiveness of the eBEfree as an innovative and suitable intervention delivery format for individuals with binge eating and overweight/obesity.

Nevertheless, the current study has important limitations. Considering the small study sample and high attrition rates, the intervention effectiveness, including the corresponding effect sizes, should be interpreted with caution. Findings generalizability should be tested in future trials with larger samples to expand the current analyzes and examine whether effects remain consistent. The choice of investigating a sub‐sample of individuals with binge eating and overweight or obesity also limits generalizability of findings, as binge eating is often present in individuals with a healthy BMI. Also, findings should be interpreted with caution, as the intervention length might be too brief to have an independent effect on BMI. It should be noted that the fact that participants provided some reasons for dropping out eBEfree related to the digital tool itself (e.g., lack of human contact; reduced interactivity; technical issues) suggests that these tools would benefit from a more blended approach (e.g., adding synchronous online sessions; adding an embedded direct channel of contact between patient and therapist). Future studies should explore the cost‐effectiveness of different approaches (app‐only, blended online, blended online and face‐to‐face). The COVID‐19 pandemic and the trial co‐occurred, which might have contributed to the observed high attrition rates at post‐intervention and at follow‐up. The feedback provided from our participants who dropped out might be helpful for future RCTs testing digital interventions for BED. The limitations of having a modest sample size in our study were attenuated by undertaking ITT analyzes comprising linear mixed models and Bayesian hierarchical models, with the latter offering a more robust approach to clinical studies with small sample sizes (van de Schoot et al. 2015). Our trial sample resulted from the application of specific eligibility criteria (absence of severe depressive symptomatology, BMI indicating overweight or obesity) that might limit generalizability of findings. Future trials will clarify the effect of the eBEfree program on more diverse samples and whether it can be used in preventive contexts. Additionally, most of the current sample was comprised of women, limiting the generalizability of the results to other gender identities. Furthermore, effects of the intervention were examined in relation to a wait list control group, which precludes conclusions regarding the effectiveness of the eBEfree in comparison to TAU or another active intervention. Future studies should conduct placebo‐controlled trials, both in terms of its format and content (e,g., a social support group mobile app for people with binge eating). Also, to assess mediating factors of intervention effectiveness on larger samples would be key to understanding how a digital integrative program produces its effect. Future studies should confirm the potential advantages of a digital intervention for binge eating, as in our study the app‐based intervention (eBEfree) showed a similar level of effectiveness to the one found in the face‐to‐face modality (BEfree) (Pinto‐Gouveia et al. 2017). Moreover, the cost‐effectiveness of this digital approach should be ascertained by comparing direct costs of running this digital tool (e.g., data server maintenance and storage) and direct expenses of TAU or specific face‐to‐face equivalent interventions (e.g., BEfree therapists fees, setting and travel expenses). Additionally, the sociodemographic background of the participants was not considered in statistical analysis. In fact, when considering RCTs of contextual‐behavioral interventions, samples are mostly composed of highly educated participants (Hughes et al. 2017), and individuals with binge eating from ethnic and gender minoritized, and economically disadvantaged groups, are underreported and overlooked in RCT studies (Burnette et al. 2022). This intersects with an ongoing discussion on the under‐representativeness and lack of generalizability of results in psychological science, which should strive for conducting research outside WEIRD (western, educated, industrialized, rich and democratic) sociodemographic groups (Henrich et al. 2010). Future studies should replicate the current study in samples with more diverse backgrounds and conduct sophisticated multi‐level analyzes where sociodemographics are explored as predictors and/or mediators of efficacy, thus contributing to tailored and patient‐centered mental health resources.

In conclusion, the current findings indicate that the eBEfree is a potentially useful tool for individuals with binge eating symptoms. The eBEfree can be presented as a timely approach that attenuates the burden of intensive resource face‐toto‐face interventions, contributing to reducing asymmetries in access to specialized interventions for disordered eating.

## Author Contributions

**Hugo Senra:** data curation, formal analysis, investigation, methodology, project administration, resources, software, validation, visualization, writing – original draft, writing – review and editing. **Cristiana Duarte:** conceptualization, funding acquisition, investigation, methodology, project administration, resources, supervision, validation, writing – original draft, writing – review and editing. **Sérgio A. Carvalho:** conceptualization, data curation, funding acquisition, investigation, methodology, resources, validation, writing – original draft, writing – review and editing. **Luís Simões:** investigation, methodology, project administration, resources. **Cláudia Ferreira:** conceptualization, funding acquisition, investigation, methodology, validation, writing – review and editing. **Lara Palmeira:** investigation, methodology, project administration, validation, writing – review and editing. **Marcela Matos:** conceptualization, investigation, methodology, validation, writing – review and editing. **Marina Cunha:** conceptualization, funding acquisition, investigation, validation, writing – review and editing. **Paula Castilho:** conceptualization, funding acquisition, investigation, validation, writing – review and editing. **Luis Cordeiro:** methodology, resources, software, validation, visualization. **Bruno Sousa:** methodology, software, validation, visualization. **José Pinto‐Gouveia:** conceptualization, funding acquisition, investigation, methodology, project administration, resources, supervision, validation, writing – review and editing.

## Conflicts of Interest

The authors declare no conflicts of interest.

## Supporting information


**Data S1.** Supporting Information.

## Data Availability

Data is available on request.

## References

[eat24432-bib-0001] Ágh, T. , G. Kovács , M. Pawaskar , D. Supina , A. Inotai , and Z. Vokó . 2015. “Epidemiology, Health‐Related Quality of Life and Economic Burden of Binge Eating Disorder: A Systematic Literature Review.” Eating and Weight Disorders‐Studies on Anorexia, Bulimia and Obesity 20, no. 1: 1–12. 10.1007/s40519-014-0173-9. PMC4349998 25571885

[eat24432-bib-0002] American Psychiatric Association . 2013. Diagnostic and Statistical Manual of Mental Disorders. 5th ed. American Psychiatric Association.

[eat24432-bib-0003] Baer, R. A. , G. T. Smith , J. Hopkins , J. Krietemeyer , and L. Toney . 2006. “Using Self‐Report Assessment Methods to Explore Facets of Mindfulness.” Assessment 13, no. 1: 27–45. 10.1177/1073191105283504. 16443717

[eat24432-bib-0005] Bates, D. , M. Mächler , B. M. Bolker , and S. C. Walker . 2015b. “Fitting Linear Mixed‐Effects Models Using lme4.” Journal of Statistical Software 67, no. 1: 1–48. 10.18637/jss.v067.i01.

[eat24432-bib-0006] Bates, D. M. , R. Kliegl , S. Vasishth , and H. Baayen . 2015a. “Parsimonious Mixed Models Douglas Bates.” arXiv E‐print, under revision.

[eat24432-bib-0007] Beck, A. T. , C. H. Ward , M. Mendelson , J. Mock , and J. Erbaugh . 1961. “An Inventory for Measuring Depression.” Archives of General Psychiatry 4, no. 6: 561. 10.1001/archpsyc.1961.01710120031004. 13688369

[eat24432-bib-0008] Bond, F. W. , S. C. Hayes , R. A. Baer , et al. 2011. “Preliminary Psychometric Properties of the Acceptance and Action Questionnaire‐II: A Revised Measure of Psychological Inflexibility and Experiential Avoidance.” Behavior Therapy 42, no. 4: 676–688. 10.1016/j.beth.2011.03.007. 22035996

[eat24432-bib-0009] Bürkner, P. C. 2017. “Brms: An R Package for Bayesian Multilevel Models Using Stan.” Journal of Statistical Software 80: 1–28. 10.18637/jss.v080.i01.

[eat24432-bib-0010] Bürkner, P. C. 2018. “Advanced Bayesian Multilevel Modeling With the R Package Brms.” R Journal 10, no. 1: 395. 10.32614/rj-2018-017.

[eat24432-bib-0011] Burnette, C. B. , J. L. Luzier , C. M. Weisenmuller , and R. L. Boutté . 2022. “A Systematic Review of Sociodemographic Reporting and Representation in Eating Disorder Psychotherapy Treatment Trials in the United States.” International Journal of Eating Disorders 55, no. 4: 423–454. 10.1002/eat.23699. 35288967 PMC8988395

[eat24432-bib-0012] Caldiroli, A. , D. La Tegola , F. Manzo , et al. 2023. “The Impact of the COVID‐19 Pandemic on Binge Eating Disorder: A Systematic Review.” Nutrients 15, no. 17: 3777. 10.3390/nu15173777. 37686811 PMC10490470

[eat24432-bib-0013] Castilho, P. , J. Pinto‐Gouveia , and J. Duarte . 2015a. “Evaluating the Multifactor Structure of the Long and Short Versions of the Self‐Compassion Scale in a Clinical Sample.” Journal of Clinical Psychology 71, no. 9: 856–870. 10.1002/jclp.22187. 25907562

[eat24432-bib-0014] Castilho, P. , J. Pinto‐Gouveia , and J. Duarte . 2015b. “Exploring Self‐Criticism: Confirmatory Factor Analysis of the FSCRS in Clinical and Nonclinical Samples.” Clinical Psychology & Psychotherapy 22, no. 2: 153–164. 10.1002/cpp.1881. 24307461

[eat24432-bib-0015] Christopher, M. S. , N. J. Neuser , P. G. Michael , and A. Baitmangalkar . 2012. “Exploring the Psychometric Properties of the Five Facet Mindfulness Questionnaire.” Mindfulness 3, no. 2: 124–131. 10.1007/s12671-011-0086-x.

[eat24432-bib-0016] Congdon, P. D. 2019. Bayesian Hierarchical Models: With Applications Using R. 2nd ed. Chapman and Hall/CRC.

[eat24432-bib-0017] Dawes, A. J. , M. Maggard‐Gibbons , A. R. Maher , et al. 2016. “Mental Health Conditions Among Patients Seeking and Undergoing Bariatric Surgery a Meta‐Analysis.” JAMA 315, no. 2: 150. 10.1001/jama.2015.18118. 26757464

[eat24432-bib-0018] di Giacomo, E. , F. Aliberti , F. Pescatore , et al. 2022. “Disentangling Binge Eating Disorder and Food Addiction: A Systematic Review and Meta‐Analysis.” Eating and Weight Disorders‐Studies on Anorexia, Bulimia and Obesity 27, no. 6: 1963–1970. 10.1007/s40519-021-01354-7. PMC9287203 35041154

[eat24432-bib-0019] Dingemans, A. , U. Danner , and M. Parks . 2017. “Emotion Regulation in Binge Eating Disorder: A Review.” Nutrients 9, no. 11: 1274. 10.3390/nu9111274. 29165348 PMC5707746

[eat24432-bib-0020] Duarte, C. , P. Gilbert , C. Stalker , et al. 2021. “Effect of Adding a Compassion‐Focused Intervention on Emotion, Eating and Weight Outcomes in a Commercial Weight Management Programme.” Journal of Health Psychology 26, no. 10: 1700–1715. 10.1177/1359105319890019. 31804147

[eat24432-bib-0021] Duarte, C. , J. Pinto‐Gouveia , and C. Ferreira . 2014. “Escaping From Body Image Shame and Harsh Self‐Criticism: Exploration of Underlying Mechanisms of Binge Eating.” Eating Behaviors 15, no. 4: 638–643. 10.1016/j.eatbeh.2014.08.025. 25248129

[eat24432-bib-0022] Duarte, C. , J. Pinto‐Gouveia , and C. Ferreira . 2015. “Expanding Binge Eating Assessment: Validity and Screening Value of the Binge Eating Scale in Women From the General Population.” Eating Behaviors 18: 41–47. 10.1016/j.eatbeh.2015.03.007. 25880043

[eat24432-bib-0023] Ferreira, C. , I. A. Trindade , C. Duarte , and J. Pinto‐Gouveia . 2015. “Getting Entangled With Body Image: Development and Validation of a New Measure.” Psychology and Psychotherapy: Theory, Research and Practice 88, no. 3: 304–316. 10.1111/papt.12047. 25409920

[eat24432-bib-0024] Ferreira, F. S. , A. Mihalik , R. A. Adams , J. Ashburner , and J. Mourao‐Miranda . 2022. “A Hierarchical Bayesian Model to Find Brain‐Behaviour Associations in Incomplete Data Sets.” NeuroImage 249: 118854. 10.1016/j.neuroimage.2021.118854. 34971767 PMC8861855

[eat24432-bib-0026] Gelman, A. , and J. Carlin . 2014. “Beyond Power Calculations: Assessing Type S (Sign) and Type M (Magnitude) Errors.” Perspectives on Psychological Science 9, no. 6: 641–651. 10.1177/1745691614551642. 26186114

[eat24432-bib-0027] Gelman, A. , J. B. Carlin , H. S. Stern , D. B. Dunson , A. Vehtari , and D. B. Rubin . 2013. Bayesian Data Analysis. 3rd ed. Chapman and Hall/CRC. 10.1201/b16018.

[eat24432-bib-0028] Gelman, A. , D. Simpson , and M. Betancourt . 2017. “The Prior Can Often Only Be Understood in the Context of the Likelihood.” Entropy 19, no. 10: 555. 10.3390/e19100555.

[eat24432-bib-0029] Giel, K. E. , C. M. Bulik , F. Fernandez‐Aranda , et al. 2022. “Binge Eating Disorder.” Nature Reviews Disease Primers 8, no. 1: 16. 10.1038/s41572-022-00344-y. PMC9793802 35301358

[eat24432-bib-0030] Gilbert, P. , M. Clarke , S. Hempel , J. N. V. Miles , and C. Irons . 2004. “Criticizing and Reassuring Oneself: An Exploration of Forms, Styles and Reasons in Female Students.” British Journal of Clinical Psychology 43, no. 1: 31–50. 10.1348/014466504772812959. 15005905

[eat24432-bib-0033] Gormally, J. , S. Black , S. Daston , and D. Rardin . 1982. “The Assessment of Binge Eating Severity Among Obese Persons.” Addictive Behaviors 7, no. 1: 47–55. 10.1016/0306-4603(82)90024-7. 7080884

[eat24432-bib-0034] Goss, K. , P. Gilbert , and S. Allan . 1994. “An Exploration of Shame Measures‐I: The Other as Shamer Scale.” Personality and Individual Differences 17, no. 5: 713–717. 10.1016/0191-8869(94)90149-X.

[eat24432-bib-0035] Gregório, S. , and J. P. Gouveia . 2011. “Facetas de mindfulness: características psicométricas de um instrumento de avaliação.” Psychologica 54, no. 54: 259–279. 10.14195/1647-8606_54_10.

[eat24432-bib-0036] Grilo, C. M. , M. A. White , V. Ivezaj , and R. Gueorguieva . 2020. “Randomized Controlled Trial of Behavioral Weight Loss and Stepped Care for Binge‐Eating Disorder: 12‐Month Follow‐Up.” Obesity 28, no. 11: 2116–2124. 10.1002/oby.22975. 32985114 PMC7644623

[eat24432-bib-0037] Grohmann, D. , and K. R. Laws . 2021. “Two Decades of Mindfulness‐Based Interventions for Binge Eating: A Systematic Review and Meta‐Analysis.” Journal of Psychosomatic Research 149: 110592. 10.1016/j.jpsychores.2021.110592. 34399197

[eat24432-bib-0038] Hayes, S. C. , K. D. Strosahl , and K. G. Wilson 1999. Acceptance and Commitment Therapy: An Experiential Approach to Behavior Change. Guilford Press.

[eat24432-bib-0039] Henrich, J. , S. J. Heine , and A. Norenzayan . 2010. “Most People Are Not WEIRD.” Nature 466, no. 7302: 29. 10.1038/466029a. 20595995

[eat24432-bib-0040] Hermanto, N. , and D. C. Zuroff . 2016. “The Social Mentality Theory of Self‐Compassion and Self‐Reassurance: The Interactive Effect of Care‐Seeking and Caregiving.” Journal of Social Psychology 156, no. 5: 523–535. 10.1080/00224545.2015.1135779. 26736073

[eat24432-bib-0041] Hilbert, A. , D. Petroff , S. Herpertz , et al. 2019. “Meta‐Analysis of the Efficacy of Psychological and Medical Treatments for Binge‐Eating Disorder.” Journal of Consulting and Clinical Psychology 87, no. 1: 91–105. 10.1037/ccp0000358. 30570304

[eat24432-bib-0042] Hilbert, A. , D. Petroff , S. Herpertz , et al. 2020. “Meta‐Analysis on the Long‐Term Effectiveness of Psychological and Medical Treatments for Binge‐Eating Disorder.” International Journal of Eating Disorders 53, no. 9: 1353–1376. 10.1002/eat.23297. 32583527

[eat24432-bib-0043] Hill, M. L. , A. Masuda , H. Melcher , J. R. Morgan , and M. P. Twohig . 2015. “Acceptance and Commitment Therapy for Women Diagnosed With Binge Eating Disorder: A Case‐Series Study.” Cognitive and Behavioral Practice 22, no. 3: 367–378. 10.1016/j.cbpra.2014.02.005.

[eat24432-bib-0044] Hughes, L. S. , J. Clark , J. A. Colclough , E. Dale , and D. McMillan . 2017. “Acceptance and Commitment Therapy (ACT) for Chronic Pain.” Clinical Journal of Pain 33, no. 6: 552–568. 10.1097/AJP.0000000000000425. 27479642

[eat24432-bib-0045] Katterman, S. N. , B. M. Kleinman , M. M. Hood , L. M. Nackers , and J. A. Corsica . 2014. “Mindfulness Meditation as an Intervention for Binge Eating, Emotional Eating, and Weight Loss: A Systematic Review.” Eating Behaviors 15, no. 2: 197–204. 10.1016/j.eatbeh.2014.01.005. 24854804

[eat24432-bib-0047] Kelly, A. C. , and J. C. Carter . 2015. “Self‐Compassion Training for Binge Eating Disorder: A Pilot Randomized Controlled Trial.” Psychology and Psychotherapy: Theory, Research and Practice 88, no. 3: 285–303. 10.1111/papt.12044. 25330466

[eat24432-bib-0048] Keski‐Rahkonen, A. 2021. “Epidemiology of Binge Eating Disorder: Prevalence, Course, Comorbidity, and Risk Factors.” Current Opinion in Psychiatry 34, no. 6: 525–531. 10.1097/YCO.0000000000000750. 34494972

[eat24432-bib-0049] Kristeller, J. L. , and R. Q. Wolever . 2010. “Mindfulness‐Based Eating Awareness Training for Treating Binge Eating Disorder: The Conceptual Foundation.” Eating Disorders 19, no. 1: 49–61. 10.1080/10640266.2011.533605. 21181579

[eat24432-bib-0050] Kristeller, J. L. W. R. Q. 2013. “Mindfulness‐Based Eating Awareness Training for Treating Binge Eating Disorder: The Conceptual Foundation.” In Eating Disorders and Mindfulness, edited by L. DeSole . Routeledge. 10.1080/10640266.2011.533605 21181579

[eat24432-bib-0051] Lammers, D. , J. Richman , J. B. Holcomb , and J. O. Jansen . 2023. “Use of Bayesian Statistics to Reanalyze Data From the Pragmatic Randomized Optimal Platelet and Plasma Ratios Trial.” JAMA Network Open 6, no. 2: e230421. 10.1001/jamanetworkopen.2023.0421. 36811858 PMC9947730

[eat24432-bib-0052] Leehr, E. J. , K. Krohmer , K. Schag , T. Dresler , S. Zipfel , and K. E. Giel . 2015. “Emotion Regulation Model in Binge Eating Disorder and Obesity—A Systematic Review.” Neuroscience & Biobehavioral Reviews 49: 125–134. 10.1016/j.neubiorev.2014.12.008. 25530255

[eat24432-bib-0053] Lee‐Winn, A. E. , L. Townsend , S. P. Reinblatt , and T. Mendelson . 2016. “Associations of Neuroticism‐Impulsivity and Coping With Binge Eating in a Nationally Representative Sample of Adolescents in the United States.” Eating Behaviors 22: 133–140. 10.1016/j.eatbeh.2016.06.009. 27289518 PMC4983245

[eat24432-bib-0054] Lemoine, N. P. 2019. “Moving Beyond Noninformative Priors: Why and How to Choose Weakly Informative Priors in Bayesian Analyses.” Oikos 128, no. 7: 912–928. 10.1111/oik.05985.

[eat24432-bib-0056] Linardon, J. , M. Messer , A. Shatte , et al. 2023. “Targeting Dietary Restraint to Reduce Binge Eating: A Randomised Controlled Trial of a Blended Internet‐ and Smartphone App‐Based Intervention.” Psychological Medicine 53, no. 4: 1277–1287. 10.1017/S0033291721002786. 34247660

[eat24432-bib-0058] Linardon, J. , A. Shatte , M. Messer , J. Firth , and M. Fuller‐Tyszkiewicz . 2020. “E‐Mental Health Interventions for the Treatment and Prevention of Eating Disorders: An Updated Systematic Review and Meta‐Analysis.” Journal of Consulting and Clinical Psychology 88, no. 11: 994–1007. 10.1037/ccp0000575. 32852971

[eat24432-bib-0060] Linardon, J. , T. D. Wade , X. De La Piedad Garcia , and L. Brennan . 2017. “The Efficacy of Cognitive‐Behavioral Therapy for Eating Disorders: A Systematic Review and Meta‐Analysis.” Journal of Consulting and Clinical Psychology 85, no. 11: 1080–1094. 10.1037/ccp0000245. 29083223

[eat24432-bib-0062] Mannucci, E. , V. Ricca , E. Barciulli , et al. 1999. “Quality of Life and Overweight.” Addictive Behaviors 24, no. 3: 345–357. 10.1016/S0306-4603(98)00055-0. 10400274

[eat24432-bib-0063] Marcus, M. D. , R. R. Wing , and D. M. Lamparski . 1985. “Binge Eating and Dietary Restraint in Obese Patients.” Addictive Behaviors 10, no. 2: 163–168. 10.1016/0306-4603(85)90022-X. 3859990

[eat24432-bib-0064] Matos, M. , J. Pinto‐Gouveia , P. Gilbert , C. Duarte , and C. Figueiredo . 2015. “The Other as Shamer Scale‐2: Development and Validation of a Short Version of a Measure of External Shame.” Personality and Individual Differences 74: 6–11. 10.1016/j.paid.2014.09.037.

[eat24432-bib-0065] Messer, M. , C. Anderson , and J. Linardon . 2021. “Self‐Compassion Explains Substantially More Variance in Eating Disorder Psychopathology and Associated Impairment Than Mindfulness.” Body Image 36: 27–33. 10.1016/j.bodyim.2020.10.002. 33161205

[eat24432-bib-0066] Mourilhe, C. , C. E. F. de Moraes , G. V. da Veiga , et al. 2021. “An Evaluation of Binge Eating Characteristics in Individuals With Eating Disorders: A Systematic Review and Meta‐Analysis.” Appetite 162: 105176. 10.1016/j.appet.2021.105176. 33639247

[eat24432-bib-0067] Neff, K. D. 2003. “The Development and Validation of a Scale to Measure Self‐Compassion.” Self and Identity 2, no. 3: 223–250. 10.1080/15298860309027.

[eat24432-bib-0068] Norder, S. J. , S. Visvalingam , P. J. Norton , and M. M. Norberg . 2023. “A Scoping Review of Psychosocial Interventions to Reduce Internalised Shame.” Psychotherapy Research 33, no. 2: 131–145. 10.1080/10503307.2022.2082340. 35706348

[eat24432-bib-0069] O'Loghlen, E. , S. Grant , and R. Galligan . 2022. “Shame and Binge Eating Pathology: A Systematic Review.” Clinical Psychology & Psychotherapy 29, no. 1: 147–163. 10.1002/cpp.2615. 34010473

[eat24432-bib-0071] Pelham, W. E., III , O. Gonzalez , S. A. Metcalf , et al. 2019. “Item Response Theory Analysis of the Five Facet Mindfulness Questionnaire and Its Short Forms.” Mindfulness 10, no. 8: 1615–1628. 10.1007/s12671-019-01105-x. 31681450 PMC6823990

[eat24432-bib-0072] Pinheiro, J. , D. Bates , S. DebRoy , D. Sarkar , S. Heisterkamp , and B. Van Willigen . 2020. Package nlme: Linear and Nonlinear Mixed Effects Models. R Package.

[eat24432-bib-0073] Pinto‐Gouveia, J. , S. A. Carvalho , L. Palmeira , et al. 2017. “BEfree: A New Psychological Program for Binge Eating That Integrates Psychoeducation, Mindfulness, and Compassion.” Clinical Psychology & Psychotherapy 24, no. 5: 1090–1098. 10.1002/cpp.2072. 28124451 PMC6686162

[eat24432-bib-0074] Pinto‐Gouveia, J. , S. A. Carvalho , L. Palmeira , et al. 2019. “Incorporating Psychoeducation, Mindfulness and Self‐Compassion in a New Programme for Binge Eating (BEfree): Exploring Processes of Change.” Journal of Health Psychology 24, no. 4: 466–479. 10.1177/1359105316676628. 27852886

[eat24432-bib-0075] Pinto‐Gouveia, J. , S. Gregório , A. Dinis , and A. Xavier . 2012. “Experiential Avoidance in Clinical and Non‐Clinical Samples: AAQ‐II Portuguese Version.” International Journal of Psychology and Psychological Therapy 12, no. 2: 139–156.

[eat24432-bib-0077] Rotella, F. , G. Fioravanti , L. Godini , E. Mannucci , C. Faravelli , and V. Ricca . 2015. “Temperament and Emotional Eating: A Crucial Relationship in Eating Disorders.” Psychiatry Research 225, no. 3: 452–457. 10.1016/j.psychres.2014.11.068. 25537489

[eat24432-bib-0078] Sala, M. , and C. A. Levinson . 2017. “A Longitudinal Study on the Association Between Facets of Mindfulness and Disinhibited Eating.” Mindfulness 8, no. 4: 893–902. 10.1007/s12671-016-0663-0. 30467917

[eat24432-bib-0079] Sala, M. , S. Shankar Ram , I. A. Vanzhula , and C. A. Levinson . 2020. “Mindfulness and Eating Disorder Psychopathology: A Meta‐Analysis.” International Journal of Eating Disorders 53, no. 6: 834–851. 10.1002/eat.23247. 32100320

[eat24432-bib-0080] Santomauro, D. F. , S. Melen , D. Mitchison , T. Vos , H. Whiteford , and A. J. Ferrari . 2021. “The Hidden Burden of Eating Disorders: An Extension of Estimates From the Global Burden of Disease Study 2019.” Lancet Psychiatry 8, no. 4: 320–328. 10.1016/S2215-0366(21)00040-7. 33675688 PMC7973414

[eat24432-bib-0081] Seixas, F. L. , B. Zadrozny , J. Laks , A. Conci , and D. C. Muchaluat Saade . 2014. “A Bayesian Network Decision Model for Supporting the Diagnosis of Dementia, Alzheimer's Disease and Mild Cognitive Impairment.” Computers in Biology and Medicine 51: 140–158. 10.1016/j.compbiomed.2014.04.010. 24946259

[eat24432-bib-0082] Serpell, L. , R. Amey , and S. K. Kamboj . 2020. “The Role of Self‐Compassion and Self‐Criticism in Binge Eating Behaviour.” Appetite 144: 104470. 10.1016/j.appet.2019.104470. 31586596

[eat24432-bib-0083] Silva, I. , J. Pais‐Ribeiro , and H. Cardoso . 2008. “Contributo para a adaptação para a população portuguesa de uma escala de avaliação da qualidade de vida específica paradoentes com obesidade: aorwelL‐97.” Psicológia, Saúde e Doencas 9, no. 1: 29–48.

[eat24432-bib-0087] Svaldi, J. , J. Griepenstroh , B. Tuschen‐Caffier , and T. Ehring . 2012. “Emotion Regulation Deficits in Eating Disorders: A Marker of Eating Pathology or General Psychopathology?” Psychiatry Research 197, no. 1–2: 103–111. 10.1016/j.psychres.2011.11.009. 22401969

[eat24432-bib-0088] Timmerman, G. M. 1999. “Binge Eating Scale: Further Assessment of Validity and Reliability1.” Journal of Applied Biobehavioral Research 4, no. 1: 1–12. 10.1111/j.1751-9861.1999.tb00051.x.

[eat24432-bib-0089] Trindade, I. A. , C. Ferreira , J. Pinto‐Gouveia , and L. Nooren . 2016. “Clarity of Personal Values and Committed Action: Development of a Shorter Engaged Living Scale.” Journal of Psychopathology and Behavioral Assessment 38, no. 2: 258–265. 10.1007/s10862-015-9509-7.

[eat24432-bib-0090] Trompetter, H. R. , P. M. Ten Klooster , K. M. G. Schreurs , M. Fledderus , G. J. Westerhof , and E. T. Bohlmeijer . 2013. “Measuring Values and Committed Action With the Engaged Living Scale (ELS): Psychometric Evaluation in a Nonclinical Sample and a Chronic Pain Sample.” Psychological Assessment 25, no. 4: 1235–1246. 10.1037/a0033813. 23914955

[eat24432-bib-0091] Udo, T. , and C. M. Grilo . 2018. “Prevalence and Correlates of DSM‐5–Defined Eating Disorders in a Nationally Representative Sample of U.S. Adults.” Biological Psychiatry 84, no. 5: 345–354. 10.1016/j.biopsych.2018.03.014. 29859631 PMC6097933

[eat24432-bib-0092] Vainik, U. , I. García‐García , and A. Dagher . 2019. “Uncontrolled Eating: A Unifying Heritable Trait Linked With Obesity, Overeating, Personality and the Brain.” European Journal of Neuroscience 50, no. 3: 2430–2445. 10.1111/ejn.14352. 30667547

[eat24432-bib-0093] van Buuren, S. , and K. Groothuis‐Oudshoorn . 2011. “Mice: Multivariate Imputation by Chained Equations in R.” Journal of Statistical Software 45, no. 3: 1–67. 10.18637/jss.v045.i03.

[eat24432-bib-0094] van de Schoot, R. , J. J. Broere , K. H. Perryck , M. Zondervan‐Zwijnenburg , and N. E. van Loey . 2015. “Analyzing Small Data Sets Using Bayesian Estimation: The Case of Posttraumatic Stress Symptoms Following Mechanical Ventilation in Burn Survivors.” European Journal of Psychotraumatology 6, no. 1: 1–13. 10.3402/ejpt.v6.25216. PMC4357639 25765534

[eat24432-bib-0095] van Zwet, E. , and A. Gelman . 2022. “A Proposal for Informative Default Priors Scaled by the Standard Error of Estimates.” American Statistician 76, no. 1: 1–9. 10.1080/00031305.2021.1938225.

[eat24432-bib-0096] Vaz Serra, A. S. 1973. “Aferição dos quadros clínicos depressivos: ensaio de aplicação do inventário depressivo de Beck a uma amostra portuguesa de doentes deprimidos.” Separata de Coimbra Médica 20: 623–644.

[eat24432-bib-0097] Villarejo, C. , F. Fernández‐Aranda , S. Jiménez‐Murcia , et al. 2012. “Lifetime Obesity in Patients With Eating Disorders: Increasing Prevalence, Clinical and Personality Correlates.” European Eating Disorders Review 20, no. 3: 250–254. 10.1002/erv.2166. 22383308 PMC3510304

[eat24432-bib-0098] Warren, J. M. , N. Smith , and M. Ashwell . 2017. “A Structured Literature Review on the Role of Mindfulness, Mindful Eating and Intuitive Eating in Changing Eating Behaviours: Effectiveness and Associated Potential Mechanisms.” Nutrition Research Reviews 30, no. 2: 272–283. 10.1017/S0954422417000154. 28718396

[eat24432-bib-0099] West, B. T. , K. Welch , A. Galecki , and B. Gillespie . 2022. Linear Mixed Models: A Practical Guide Using Statistical Software. Chapman and Hall/CRC.

[eat24432-bib-0100] Williams, B. M. , and C. A. Levinson . 2022. “A Model of Self‐Criticism as a Transdiagnostic Mechanism of Eating Disorder Comorbidity: A Review.” New Ideas in Psychology 66: 100949. 10.1016/j.newideapsych.2022.100949.

[eat24432-bib-0101] Wilson, D. R. , N. J. Loxton , and A. O'Donovan . 2021. “From BIS to Binge: The Role of Negative Affect in the Pathway Between Personality and Binge Eating.” Eating Behaviors 41: 101479. 10.1016/j.eatbeh.2021.101479. 33631490

[eat24432-bib-0102] World Health Organization . 2023. “Advancing Access to Affordable, Quality Mental Health Services Through a Systems‐Strengthening Approach.” https://www.who.int/news‐room/feature‐stories/detail/advancing‐access‐to‐affordable‐‐quality‐mental‐health‐services‐through‐a‐systems‐strengthening‐approach.

[eat24432-bib-0103] World Health Organization United Nations Children's Fund (UNICEF) . 2018. “A Vision for Primary Health Care in the 21st Century: Towards Universal Health Coverage and the Sustainable Development Goals.” https://iris.who.int/handle/10665/328065.

