# Supplementary material for: eBEfree: Combining Psychoeducation, Mindfulness, and Self‐Compassion in an App‐Based Psychological Intervention to Manage Binge‐Eating Symptoms: A Randomized Controlled Trial
Source: Int J Eat Disord. 2025 Mar 29;58(7):1199–218. doi: 10.1002/eat.24432 (PMC12232357; doi:10.1002/eat.24432)
Supplement: Supplementary file 1 — Data S1. Supporting Information. [file EAT-58-1199-s001.pdf]

SUPPLMENTAL MATERIALS

CONSORT 2010 checklist of information to include when reporting a randomised trial*

Section/Topic	Item No	Checklist item	Reported on page No
**Title and abstract**			
	1a	Identification as a randomised trial in the title	1
	1b	Structured summary of trial design, methods, results, and conclusions (for specific guidance see CONSORT for abstracts)	3
**Introduction**			
Background and objectives	2a	Scientific background and explanation of rationale	4-7
	2b	Specific objectives or hypotheses	6,7
**Methods**			
Trial design	3a	Description of trial design (such as parallel, factorial) including allocation ratio	7,8
	3b	Important changes to methods after trial commencement (such as eligibility criteria), with reasons	7
Participants	4a	Eligibility criteria for participants	8,9
	4b	Settings and locations where the data were collected	8,9
Interventions	5	The interventions for each group with sufficient details to allow replication, including how and when they were actually administered	10,11
Outcomes	6a	Completely defined pre-specified primary and secondary outcome measures, including how and when they were assessed	11-15
	6b	Any changes to trial outcomes after the trial commenced, with reasons	7
Sample size	7a	How sample size was determined	15,16
	7b	When applicable, explanation of any interim analyses and stopping guidelines	16-18
Randomisation:			
Sequence generation	8a	Method used to generate the random allocation sequence	9,10
	8b	Type of randomisation; details of any restriction (such as blocking and block size)	9,10
Allocation concealment mechanism	9	Mechanism used to implement the random allocation sequence (such as sequentially numbered containers), describing any steps taken to conceal the sequence until interventions were assigned	9,10
Implementation	10	Who generated the random allocation sequence, who enrolled participants, and who assigned participants to interventions	9,10
Blinding	11a	If done, who was blinded after assignment to interventions (for example, participants, care providers, those assessing outcomes) and how	9,10

	11b	If relevant, description of the similarity of interventions	16-18
Statistical methods	12a	Statistical methods used to compare groups for primary and secondary outcomes	16-18
	12b	Methods for additional analyses, such as subgroup analyses and adjusted analyses	17,18
**Results**			
Participant flow (a diagram is strongly recommended)	13a	For each group, the numbers of participants who were randomly assigned, received intended treatment, and were analysed for the primary outcome	18,19
	13b	For each group, losses and exclusions after randomisation, together with reasons	18,19
Recruitment	14a	Dates defining the periods of recruitment and follow-up	8
	14b	Why the trial ended or was stopped	8,9
Baseline data	15	A table showing baseline demographic and clinical characteristics for each group	18 (table 1)
Numbers analysed	16	For each group, number of participants (denominator) included in each analysis and whether the analysis was by original assigned groups	19 (Figure 1)
Outcomes and estimation	17a	For each primary and secondary outcome, results for each group, and the estimated effect size and its precision (such as 95% confidence interval)	19 (Table 2)
	17b	For binary outcomes, presentation of both absolute and relative effect sizes is recommended	N/A
Ancillary analyses	18	Results of any other analyses performed, including subgroup analyses and adjusted analyses, distinguishing pre-specified from exploratory	19-21
Harms	19	All important harms or unintended effects in each group (for specific guidance see CONSORT for harms)	19-21
**Discussion**			
Limitations	20	Trial limitations, addressing sources of potential bias, imprecision, and, if relevant, multiplicity of analyses	23,24
Generalisability	21	Generalisability (external validity, applicability) of the trial findings	21-23
Interpretation	22	Interpretation consistent with results, balancing benefits and harms, and considering other relevant evidence	21-23
**Other information**			
Registration	23	Registration number and name of trial registry	8
Protocol	24	Where the full trial protocol can be accessed, if available	8
Funding	25	Sources of funding and other support (such as supply of drugs), role of funders	Suppl Materials

Citation: Schulz KF, Altman DG, Moher D, for the CONSORT Group. CONSORT 2010 Statement: updated guidelines for reporting parallel group randomised trials. BMC Medicine. 2010;8:18.
© 2010 Schulz et al. This is an Open Access article distributed under the terms of the Creative Commons Attribution License (http://creativecommons.org/licenses/by/2.0), which permits unrestricted use, distribution, and reproduction in any medium, provided the original work is properly cited.

*We strongly recommend reading this statement in conjunction with the CONSORT 2010 Explanation and Elaboration for important clarifications on all the items. If relevant, we also recommend reading CONSORT extensions for cluster randomised trials, non-inferiority and equivalence trials, non-pharmacological treatments, herbal interventions, and pragmatic trials. Additional extensions are forthcoming: for those and for up-to-date references relevant to this checklist, see www.consort-statement.org.

PRIMARY AND SECONDARY OUTCOME MEASURES INCLUDED IN THE TRIAL

Primary Outcome

The primary outcome – binge eating symptomatology - was collected using an online standardized questionnaire, the Binge-Eating Scale (BES) (Duarte et al., 2015; Gormally et al., 1982). The BES is a self-reported measure comprising 16 items that address key behavioural, affective, and cognitive symptoms associated with binge eating (e.g. rapid eating, eating large amounts of food in a short period of time, uncontrolled eating behaviours, binge-eating related guilt). BES total score ranges from 0 to 46 and higher scores reflect higher severity of binge eating symptoms. Scores ranging from 0 to 17 suggest no or minimal binge-eating, 18 to 26 suggest mild to moderate binge-eating, and scores equal or greater than 27 suggest severe binge-eating. The Portuguese version of BES showed an internal consistency (α) of 0.88 (Duarte et al., 2015).

Secondary Outcomes

Depression. Beck Depression Inventory (BDI) (Beck et al., 1961; Vaz Serra, 1973). BDI is a well-known 21-item questionnaire (with a 4-point Likert scale) that measures current depressive symptoms. The final score ranges from 1 to 40 and suggest clinical depression when it is greater than 20. The Portuguese version shows similar psychometric properties (Vaz Serra, 1973). In the current study, BDI presented an internal consistency of $\alpha = 0.92$.

Self-Compassion. Self-Compassion Scale (SCS) (Castilho et al., 2015a; NEFF, 2003) includes 26 items belonging to six subscales, with three subscales standing for self-compassion – self-kindness (e.g., "I try to be loving towards myself when I'm feeling emotional pain"); common humanity (e.g., "When things are going badly for me, I see the difficulties as part of life that everyone goes through"), and mindfulness (e.g., "When something painful happens I try to take a balanced view of the situation") – and another three subscales representing self-judgement (e.g., "When times are really difficult, I tend to be tough on myself"), isolation (e.g., "When I'm really struggling, I tend to feel like other people must be having an easier time of it") and over-identification (e.g., "When something painful happens I tend to blow the incident out of proportion"). Participants respond according to a 5-point Likert scale (1 = *almost never*; 5 = *almost always*). Greater scores in each subscale suggest more self-compassion, with the subscales *Self-Judgment, Isolation*, and *Overidentification* being reverse coded. In the Portuguese version of the scale, Cronbach's alphas (α) ranged from 0.92 to 0.94 for the total scale, and from 0.70 to 0.88 for the subscales (Castilho et al., 2015a).

Mindfulness. Five Facet Mindfulness Questionnaire-15 (FFMQ-15) (Baer et al., 2006; Gregório & Gouveia, 2011). The instrument is composed of 15 items and is the shorter version of the original 39 items questionnaire (e.g., "I find myself doing things without paying attention"; "When I have distressing thoughts or images I am able just to notice them without reacting"). FFMQ-15 measures the dispositional and multifaceted mindfulness characteristics. Participants are asked to rate how mindful they feel in daily life in a 5-point Likert scale (1 = *never or very rarely true*; 5 = *very often or always true*). FFMQ-15 presents the same five-factor structure as the original version, as well as good internal consistency in the Portuguese version of the instrument (α ranging from 0.66 to 0.89) (Gregório & Gouveia, 2011).

Psychological flexibility. The Acceptance and Action Questionnaire-II (AAQ-II) (Bond et al., 2011; Pinto-Gouveia et al., 2012) is a seven-item self-report questionnaire that assesses psychological inflexibility, with items being scored on a Likert-type scale (1 = '*never*' to 7 = '*always*'). AAQ-II addresses different dimensions of psychological inflexibility, including tendencies to make negative evaluations of private events and the unwillingness to be in contact with private events (e.g., "I'm afraid of my feelings' and 'my painful memories prevent me from having a fulfilling life"). A higher total score suggests greater levels of psychological inflexibility. The Portuguese version of AAQ-II showed an internal consistency (α) of 0.89 (Pinto-Gouveia et al., 2012).

Wellbeing. The Obesity Related Well-being–Revised (ORWELL-97) (Mannucci et al., 1999; Silva et al., 2008). The ORWELL-97 is an 18-item self-report instrument designed to address obesity-related wellbeing in the domains of physical symptoms, physical discomfort, and familial, social, and occupational functioning. In the questionnaire the frequency and severity of symptoms, and the extent to which certain domains are important in relation to obesity are rated on a 4-point scale from 0 (*not at all*) to 3 (*much*) (e.g., "Does shortness of breath represent an obstacle for your daily activities?"; "How important is it for you to exercise regularly?"). Higher scores indicate lower obesity-related quality of life. A good internal consistency was found in the Portuguese version of the instrument (α = 0.85) (Silva et al., 2008).

Self-criticism. Forms of Self-Criticising / Attacking & Self- Reassuring Scale (FSCRS) (Castilho et al., 2015b; Gilbert et al., 2004) is a 22-item self-report scale of forms of self-criticism (inadequate-self, which focuses on a sense of personal inadequacy (e.g., "I am easily disappointed with myself"), and hated-self, which measures the desire to hurt or persecute the self (e.g. "I call myself names") and the ability to self-reassure when things go wrong (e.g. "I

find it easy to forgive myself"). The scale is scored on a five-point Likert scale ranging from 0 ("*not at all like me*"), to 4 ("*extremely like me*"). In the Portuguese version of the scale (Castilho et al., 2015b) the Cronbach's alphas for the non-clinical sample were of 0.90 for inadequate-self and 0.85 for both the hated-self and the reassured-self. For the clinical sample Cronbach's alphas (α) were of 0.91 for the inadequate-self, 0.87 for the hated-self, and 0.85 for the reassured-self.

Shame. Other as Shamer Scale (OSS) (Goss et al., 1994; Matos et al., 2015) is a 5-point Likert scale composed of 18 items (e.g., "I feel other people see me as not good enough", "other people put me down a lot") that assesses the perception of being negatively evaluated by others. Higher scores indicate higher external shame (Goss et al., 1994). OSS has consistently showed high internal consistency, both in clinical and nonclinical samples ($\alpha = 0.96$ and 0.92, respectively) (Goss et al., 1994). Similar results were found for the Portuguese version ($\alpha = 0.91$) (Matos et al., 2015).

Body image cognitive fusion. Cognitive Fusion Questionnaire-Body Image (CFQ-BI) (Ferreira et al., 2015) is a self-report questionnaire based on the original CFQ (Gillanders et al., 2014). The questionnaire includes 10 items measuring cognitive fusion related to body image. Participants are asked to rate the extent to which each statement (e.g., "My thoughts relating to my body image cause me great distress or emotional pain") is true regarding their own experience, using a 7-point Likert scale (1 = *Never true*; 7 = *Always true*). The Portuguese version of the instrument showed good internal consistency, retest reliability, discriminant, convergent and divergent validities, with a α of 0.90 (Ferreira et al., 2015) .

Values-based behaviour. The Engaged Living Scale (ELS) (Trindade et al., 2016; Trompetter et al., 2013) is a self-report measure that assesses engagement with value-driven behaviour. The measure comprises 16 items to be rated by participants on a 5-point Likert scale (1 = *completely disagree*; 5 = *completely agree*). Higher scores express increased clarity and engagement with personal values and greater life fulfilment (e.g., "I have values that give my life more meaning"; "I feel that I am living a full life"). Previous studies with Portuguese samples with the scale and with a shorter version of it, showed good internal consistency, $\alpha = 0.88$, and $\alpha = 0.86$ respectively (Trindade et al., 2016).

References:

Baer, R. A., Smith, G. T., Hopkins, J., Krietemeyer, J., & Toney, L. (2006). Using self-report assessment methods to explore facets of mindfulness. *Assessment, 13*(1). https://doi.org/10.1177/1073191105283504

Beck, A. T., Ward, C. H., Mendelson, M., Mock, J., & Erbaugh, J. (1961). An Inventory for Measuring Depression. *Archives of General Psychiatry*, *4*(6). https://doi.org/10.1001/archpsyc.1961.01710120031004

Bond, F. W., Hayes, S. C., Baer, R. A., Carpenter, K. M., Guenole, N., Orcutt, H. K., Waltz, T., & Zettle, R. D. (2011). Preliminary Psychometric Properties of the Acceptance and Action Questionnaire-II: A Revised Measure of Psychological Inflexibility and Experiential Avoidance. *Behavior Therapy*, *42*(4). https://doi.org/10.1016/j.beth.2011.03.007

Castilho, P., Pinto-Gouveia, J., & Duarte, J. (2015a). Evaluating the Multifactor Structure of the Long and Short Versions of the Self-Compassion Scale in a Clinical Sample. *Journal of Clinical Psychology*, *71*(9). https://doi.org/10.1002/jclp.22187

Castilho, P., Pinto-Gouveia, J., & Duarte, J. (2015b). Exploring Self-criticism: Confirmatory Factor Analysis of the FSCRS in Clinical and Nonclinical Samples. *Clinical Psychology and Psychotherapy*, *22*(2). https://doi.org/10.1002/cpp.1881

Duarte, C., Pinto-Gouveia, J., & Ferreira, C. (2015). Expanding binge eating assessment: Validity and screening value of the Binge Eating Scale in women from the general population. *Eating Behaviors*, *18*. https://doi.org/10.1016/j.eatbeh.2015.03.007

Ferreira, C., Trindade, I. A., Duarte, C., & Pinto-Gouveia, J. (2015). Getting entangled with body image: Development and validation of a new measure. *Psychology and Psychotherapy: Theory, Research and Practice*, *88*(3). https://doi.org/10.1111/papt.12047

Gilbert, P., Clarke, M., Hempel, S., Miles, J. N. V., & Irons, C. (2004). Criticizing and reassuring oneself: An exploration of forms, styles and reasons in female students. *British Journal of Clinical Psychology*, *43*(1). https://doi.org/10.1348/014466504772812959

Gillanders, D. T., Bolderston, H., Bond, F. W., Dempster, M., Flaxman, P. E., Campbell, L., Kerr, S., Tansey, L., Noel, P., Ferenbach, C., Masley, S., Roach, L., Lloyd, J., May, L., Clarke, S., & Remington, B. (2014). The Development and Initial Validation of the Cognitive Fusion Questionnaire. *Behavior Therapy*, *45*(1). https://doi.org/10.1016/j.beth.2013.09.001

Gormally, J., Black, S., Daston, S., & Rardin, D. (1982). The assessment of binge eating severity among obese persons. *Addictive Behaviors*, *7*(1). https://doi.org/10.1016/0306-4603(82)90024-7

Goss, K., Gilbert, P., & Allan, S. (1994). An exploration of shame measures-I: The other as Shamer scale. *Personality and Individual Differences*, *17*(5). https://doi.org/10.1016/0191-8869(94)90149-X

Gregório, S., & Gouveia, J. P. (2011). Facetas de mindfulness: características psicométricas de um instrumento de avaliação. *Psychologica*, *54*. https://doi.org/10.14195/1647-8606_54_10

Mannucci, E., Ricca, V., Barciulli, E., Di Bernardo, M., Travaglini, R., Cabras, P. L., & Rotella, C. M. (1999). Quality of life and overweight: The Obesity Related Well-Being (ORWELL 97) questionnaire. *Addictive Behaviors*, *24*(3). https://doi.org/10.1016/S0306-4603(98)00055-0

Matos, M., Pinto-Gouveia, J., Gilbert, P., Duarte, C., & Figueiredo, C. (2015). The Other As Shamer Scale - 2: Development and validation of a short version of a measure of external shame. *Personality and Individual Differences*, *74*. https://doi.org/10.1016/j.paid.2014.09.037

NEFF, K. D. (2003). The Development and Validation of a Scale to Measure Self-Compassion. *Self and Identity*, *2*(3). https://doi.org/10.1080/15298860309027

Pinto-Gouveia, J., Gregório, S., Dinis, A., & Xavier, A. (2012). Experiential avoidance in clinical and non-clinical samples: AAQ-II Portuguese version. *International Journal of Psychology and Psychological Therapy*, *12*(2).

Silva, I., Pais-Ribeiro, J., & Cardoso, H. (2008). CONTRIBUTO PARA A ADAPTAÇÃO PARA A POPULAÇÃO PORTUGUESA DE UMA ESCALA DE AVALIAÇÃO DA QUALIDADE DE VIDA ESPECÍFICA PARADOENTES COM OBESIDADE: AORWELL-97. *Psicológia, Saúde e Doencas*, *9*(1).

Trindade, I. A., Ferreira, C., Pinto-Gouveia, J., & Nooren, L. (2016). Clarity of Personal Values and Committed Action: Development of a Shorter Engaged Living Scale. *Journal of Psychopathology and Behavioral Assessment*, *38*(2). https://doi.org/10.1007/s10862-015-9509-7

Trompetter, H. R., Ten Klooster, P. M., Schreurs, K. M. G., Fledderus, M., Westerhof, G. J., & Bohlmeijer, E. T. (2013). Measuring values and committed action with the engaged living scale (ELS): Psychometric evaluation in a nonclinical sample and a chronic pain sample. *Psychological Assessment*, *25*(4). https://doi.org/10.1037/a0033813

Vaz Serra, A. S. , A. J. L. (1973). Aferição dos quadros clínicos depressivos: ensaio de aplicação do inventário depressivo de Beck a uma amostra portuguesa de doentes deprimidos. *Separata de Coimbra Médica*, *20*, 623–644.

EBEFREE PROGRAMME STRUCTURE

Programme Session	Main Topics Covered	Content Format	Recommended duration	Between-sessions tasks
1	Introduction: generating creative hopelessness; promoting willingness.<br><br>Session to introduce the eBEfree programme, including key concepts related to binge-eating, and strategies adopted to cope with binge-eating behaviours, such as mindful eating.	Texts addressing key concepts and Videos (animated videos and videos with therapists) explaining the programme and discussing strategies to cope with binge-eating.	1 week	Mindful eating practice.
2 and 3	Psychoeducation: tackling eating misconceptions (e.g. inflexible dietary plans); clarifying the importance of physical exercise and of balanced meals; clarifying evolutionary aspects of eating behaviour; explaining the function of BE as an affect regulation strategy.	Texts addressing key concepts and practical mindfulness exercises and a questionnaire about ongoing strategies to cope with binge-eating; Audios with mindfulness exercises; and Videos (animated videos and videos with therapists) dedicated to psychoeducation.	2 weeks	Mindful-based activities (including mindful eating)
4	Emotional awareness and emotional regulation. Main goals include promoting emotional awareness and emotion regulation skills and strategies and link them to potential triggers of binge-eating behaviours.	Texts with emotional-awareness and emotional regulation-based exercises; A questionnaire about the emotional regulation exercises; videos with therapists addressing emotional regulation; audios for mindfulness exercises.	1 week	Mindful-based activities (including mindful eating)

5	Motives and Values: clarification of health-focused and self-to-self relating values. Main goals: to discuss the relationship between individual motivations and affect regulation, what values are, and the difference between values and objectives.	Texts with exercises inviting to think about values and objectives; A questionnaire about the emotional regulation exercises; videos with therapists addressing emotional regulation; videos with therapists and animated videos about values and objectives; audios for mindfulness exercises.	1 week	Mindful-based activities (including mindful eating)
6	From Fusion to Openness: promoting cognitive defusion and willingness. Main goals: to promote a better understanding of how our mind works when dealing with emotional distress; to discuss how language is important in relation to our thoughts, emotions and behaviours.	Texts with practical exercises for promoting cognitive defusion and willingness (e.g. naming our experiences and feelings); videos with therapists exploring cognitive defusion-related concepts; audios with mindfulness practices.	1 week	Mindful-based activities (including mindful eating)
7	Acceptance. Main goals: to promote self-acceptance and willingness to experience negative thought and emotions; introduction to acceptance as alternative to avoidance.	Texts with exercises addressing topics related to acceptance; Video with therapist about willingness to experience negative emotions; audios with mindfulness-based exercises.	1 week	Mindful-based activities (including mindful eating)
8 and 9	Mindfulness: developing mindfulness skills through mindfulness meditation practices including breathing meditation, body scan, meditation of thoughts, mindful eating.	Texts, audios and videos with mindfulness exercises, such as breathing, body-scan, thinking, and mindful eating;<br>Questionnaires about the experience of practicing mindfulness.	2 weeks	Mindful-based activities (including mindful breathing and self-acceptance)

10 and 11	Compassion: clarifying the negative impact of getting entangled with self-criticism; promoting self-compassion through meditation practices and compassionate imagery, including loving-kindness, safe place, compassionate figure meditation.	Texts and videos explaining the concept of self-compassion and its importance to promote self-acceptance, emotional regulation and mindfulness-based skills, and promoting self-compassion exercises; Questionnaires about the experience of practicing self-compassion.	2 weeks	Mindfulness-based and self-compassion-based exercises.
12	Committed action: promoting actions that are congruent with personal life values.	Texts, audios and videos to practice self-compassion and mindfulness based exercises, and to promote a personal commitment with a set of actions and goals related to the skills and concepts developed throughout the programme;<br><br>Setting key goals for the future, including to keep practicing mindfulness and self-compassion to help managing binge-eating behaviours and negative thoughts.	1 week	Mindfulness-based and self-compassion-based exercises.

Power Calculations – eBEfree Trial

Software: G-Power 3.1.9.7

**F tests** – ANOVA: Repeated measures, within-between interaction			
**Analysis:**	A priori: Compute required sample size		
**Input:**	Effect size f	=	0.25
	α err prob	=	0.05
	Power (1–β err prob)	=	0.95
	Number of groups	=	2
	Number of measurements	=	3
	Corr among rep measures	=	0.5
	Nonsphericity correction ϵ	=	1
**Output:**	Noncentrality parameter λ	=	16.5000000
	Critical F	=	3.1051566
	Numerator df	=	2.0000000
	Denominator df	=	84.0000000
	Total sample size	=	44

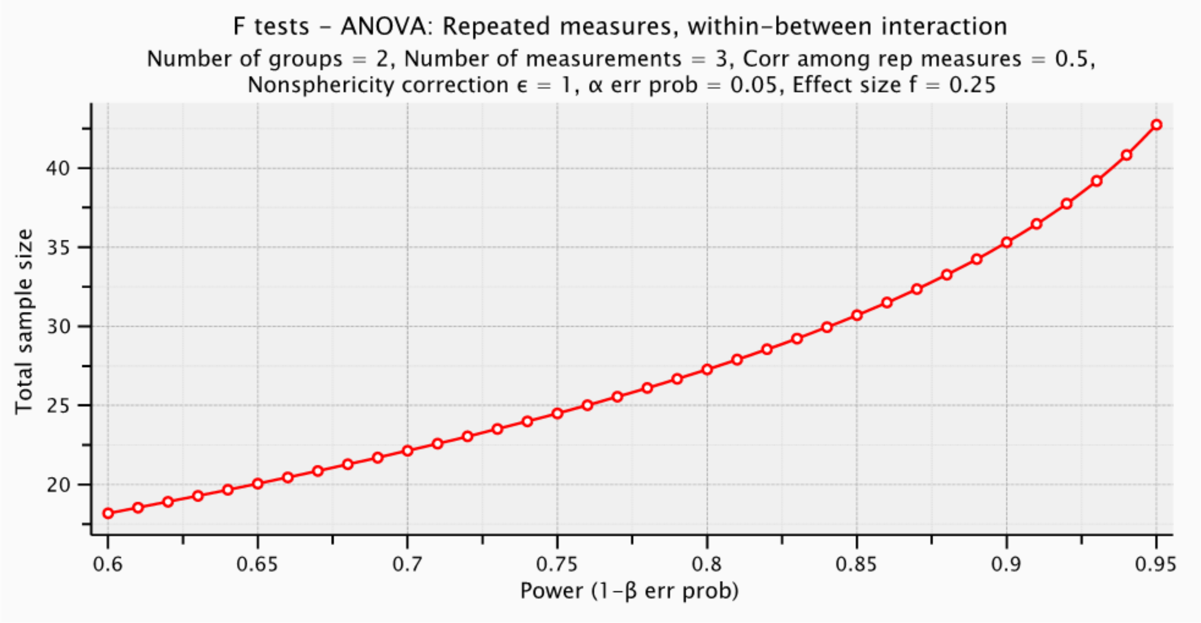

PARTICIPANTS' FEEDBACK ON THE EBEFREE PROGRAMME

QUESTION	N (%)
**Questions about the Programme's duration**	
The programme was too short	
Yes	2 (7%)
No	18 (62%)
Neutral	9 (31%)
The programme's length was adequate	
Yes	15 (52%)
No	4 (14%)
Neutral	10 (34%)
The programme was too long	
Yes	2 (7%)
No	19 (66%)
Neutral	8 (27%)
Sessions were too short	
Yes	1 (3%)
No	22 (76%)
Neutral	6 (21%)
Adequate sessions' length	
Yes	24 (83%)
No	0
Neutral	5 (17%)
Sessions were too long	
Yes	0
No	27 (93%)
Neutral	2 (7%)
**Questions about the app's usability**	
Preferred sessions' content	
Audios	24
Videos	15
Animated Videos	20
Texts	8
Interactive content	6
I Would like to use the app frequently	
Yes	19 (66%)
No	4 (14%)
Neutral	6 (20%)
I found the eBEfree app too complex	
Yes	4 (14%)
No	4 (14%)
Neutral	21 (72%)
I found the eBEfree app easy to use	
Yes	25 (86%)
No	2 (7%)
Neutral	2 (7%)
I found the eBEfree app difficult to use	
Yes	1 (3%)
No	28 (97%)

Neutral	0
I think I would need technical support to use the eBEfree app	
Yes	0
No	27 (93%)
Neutral	2 (7%)
I found the eBEfree app features well integrated	
Yes	25 (86%)
No	3 (11%)
Neutral	1 (3%)
I found the eBEfree app to have several inconsistencies	
Yes	3 (11%)
No	22 (85%)
Neutral	4 (14%)
I found the eBEfree app easy to use by most people	
Yes	29 (100%)
No	0
Neutral	0
I felt confident when using the eBEfree app	
Yes	24 (83%)
No	0
Neutral	5 (17%)
There was a long learning curve to use the eBEfree app	
Yes	0
No	0
Neutral	29 (100%)
I Would like to use the app frequently	
Yes	19 (66%)
No	4 (14%)
Neutral	6 (20%)
**Questions about the Programme's content**	
Please rate the quality of the app audios	
Positive	26 (89%)
Negative	0
Neutral	3 (11%)
Please rate the quality of the app videos	
Positive	24 (83%)
Negative	0
Neutral	5 (17%)
Please rate the quality of the app animated videos	
Positive	25 (86%)
Negative	1 (3%)
Neutral	3 (11%)
Please rate the quality of the app texts	
Positive	27 (93%)
Negative	0

Neutral	2 (7%)
Please rate the quality of the app interactive content	
Positive	23 (80%)
Negative	1 (3%)
Neutral	5 (17%)
How often did you use the app's forum chat	
Never	22 (85%)
Occasionally	7 (15%)
Often	0
How helpful did you find the app's forum chat	
Not helpful	15 (52%)
Little helpful	12 (41%)
Moderately helpful	2 (7%)
Very helpful	0
Programme's sessions found to be most helpful	
1	10
2	13
3	10
4	11
5	8
6	7
7	11
8	17
9	13
10	16
11	13
12	13
**Questions about the Programme's practice and exercises**	
On average how often did I practice mindfulness and self-compassion exercises during the programme	
Never	2 (7%)
Once a week	17 (59%)
2 times a week	3 (10%)
3 times a week	5 (17%)
5 times a week	1 (3.5%)
Everyday	1 (3.5%)
Practice of mindfulness and self-compassion exercises during the programme	
When waking up	4
Middle morning	4
Before lunch	0
At lunch time	2
After lunch	1
Middle afternoon	7
End of the day	3
At dinner time	1

After dinner	7
At night when going to sleep	9
How helpful did you find the audios with mindfulness and self-compassion exercises	
Not helpful	2 (7%)
Little helpful	5 (17%)
Moderately helpful	7 (24%)
Very helpful	15 (52%)
How likely you are to continue practicing mindfulness and self-compassion exercises (after the eBEfree programme)	
Unlikely	1 (3%)
Little likely	8 (28%)
Likely	7 (24%)
Very likely	6 (21%)
For sure	7 (24%)
**Questions on the Programme's therapeutic impact**	
After completing the eBEfree programme I find my difficulties to be	
Worse than before	0
Same as before	8 (28%)
Improved	18 (62%)
Much improved	3 (10%)
Since I started the eBEfree programme my way of coping with difficulties has got	
Worse than before	0
Same as before	6 (21%)
Improved	20 (69%)
Much improved	3 (10%)
Since I started the eBEfree programme my life in general has got	
Worse than before	0
Same as before	9 (31%)
Improved	18 (62%)
Much improved	2 (7%)
The programme helped me to be less self-critical	
Didn't help me at all	2 (7%)
Helped only a little	9 (31%)
Helped moderately	11 (38%)
Helped a lot	7 (24%)
The programme helped me to accept the idea that my overweight / obesity shouldn't affect my life	
Didn't help me at all	4 (14%)
Helped only a little	5 (17%)
Helped moderately	13 (45%)
Helped a lot	7 (24%)

The programme helped me to feel less stigma in relation to my weight	
Didn't help me at all	7 (24%)
Helped only a little	3 (7%)
Helped moderately	11 (38%)
Helped a lot	8 (28%)
The programme helped me to feel more self-compassionate in relation to myself and my own difficulties	
Didn't help me at all	5 (17%)
Helped only a little	3 (10%)
Helped moderately	9 (31%)
Helped a lot	12 (41%)
The programme helped me to find a new life purpose and new values	
Didn't help me at all	6 (21%)
Helped only a little	8 (28%)
Helped moderately	10 (34%)
Helped a lot	5 (17%)
The programme helped me to figure out the dangers of being at the "automatic pilot"	
Didn't help me at all	2 (7%)
Helped only a little	6 (20%)
Helped moderately	13 (45%)
Helped a lot	8 (28%)
The programme helped me to live more in the present	
Didn't help me at all	3 (10%)
Helped only a little	4 (14%)
Helped moderately	8 (28%)
Helped a lot	14 (48%)
The programme helped me to not get stuck to what my mind tells me about my weight	
Didn't help me at all	5 (17%)
Helped only a little	4 (14%)
Helped moderately	11 (38%)
Helped a lot	9 (31%)
The programme helped me to be more aware of my own emotions	
Didn't help me at all	1 (3.5%)
Helped only a little	5 (17.5%)
Helped moderately	12 (41%)
Helped a lot	11 (38%)
The programme helped me to cope better with my own emotions	
Didn't help me at all	4 (14%)
Helped only a little	6 (20%)
Helped moderately	11 (38%)
Helped a lot	8 (28%)

EBEFREE APP ILLUSTRATIVE SCREEN SHOTS

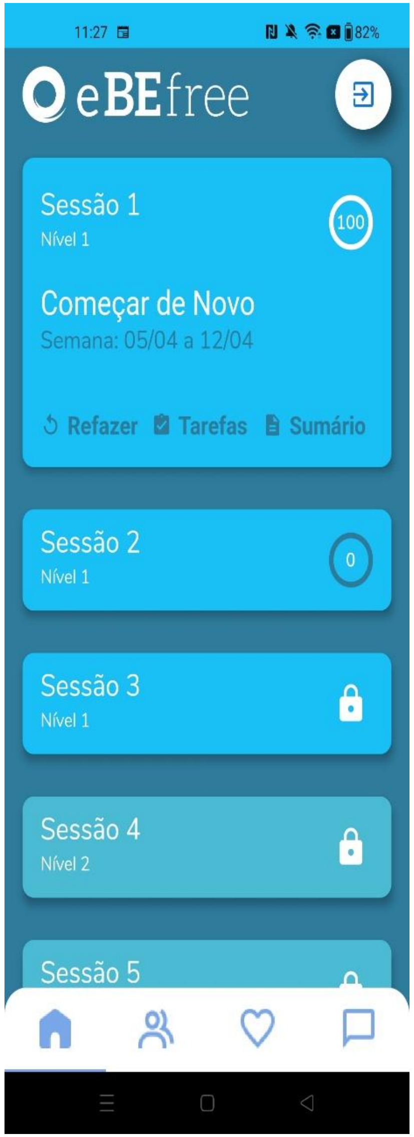

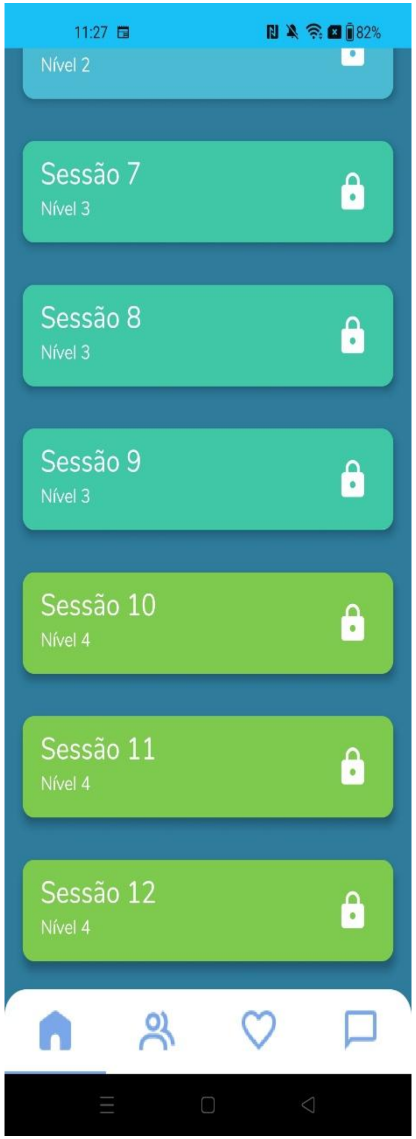

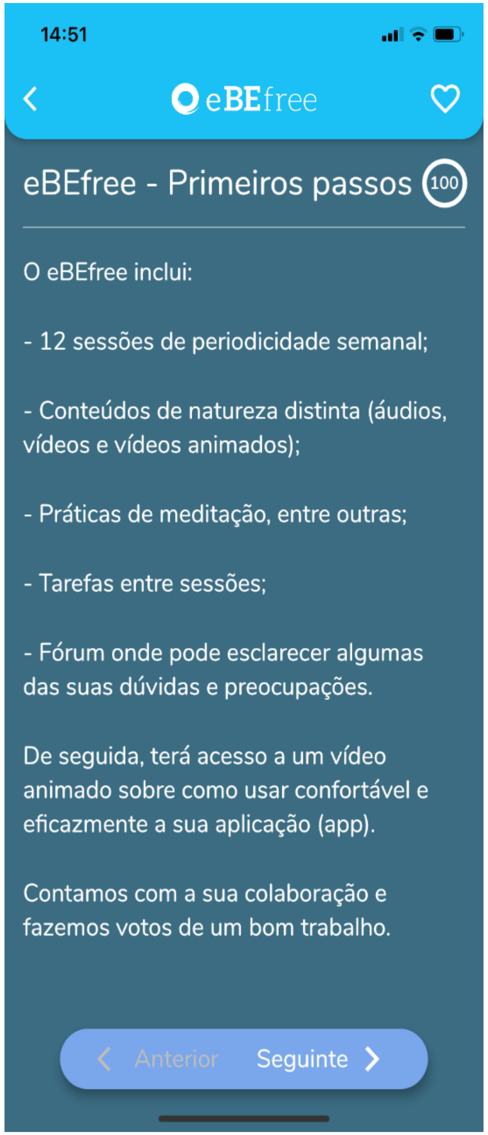

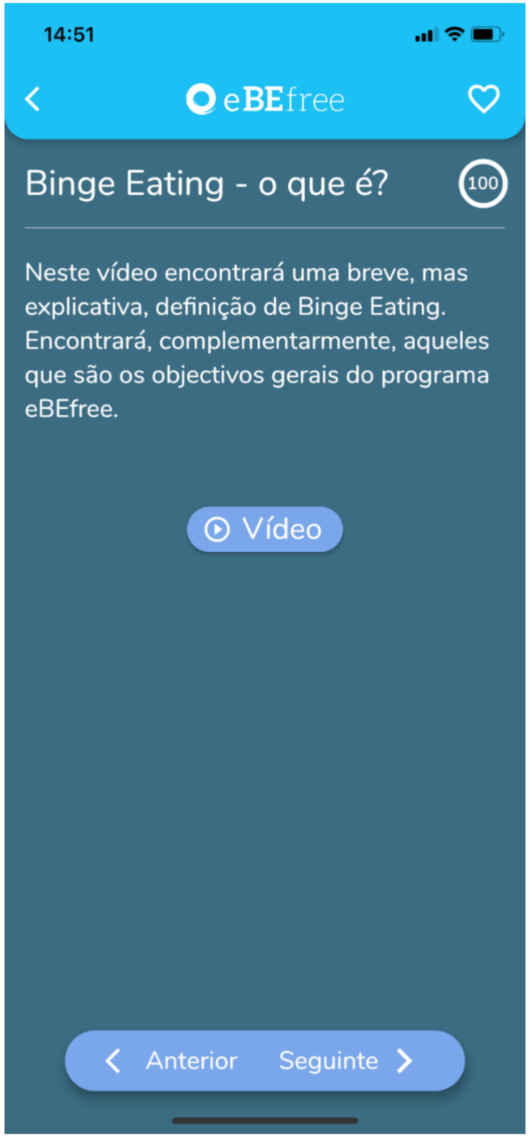

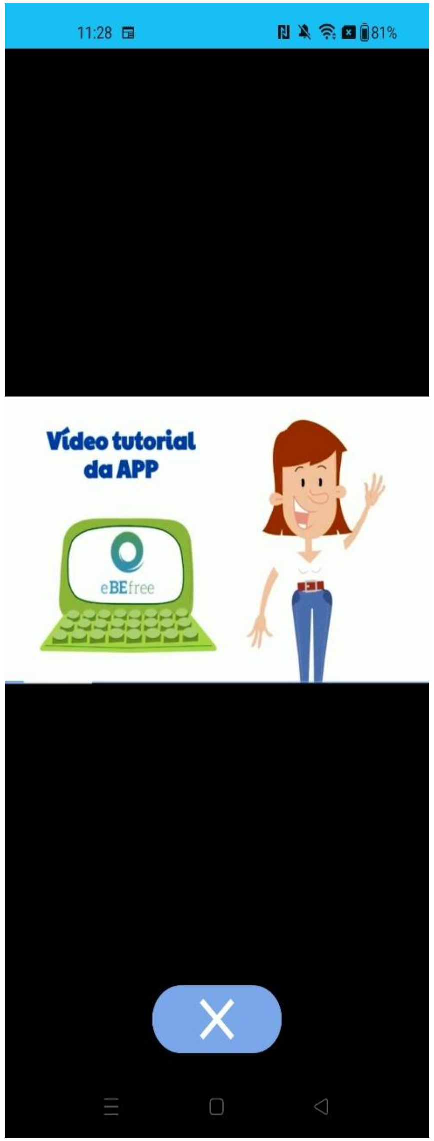
11:28
81%
Vídeo tutorial da APP
eBEfree

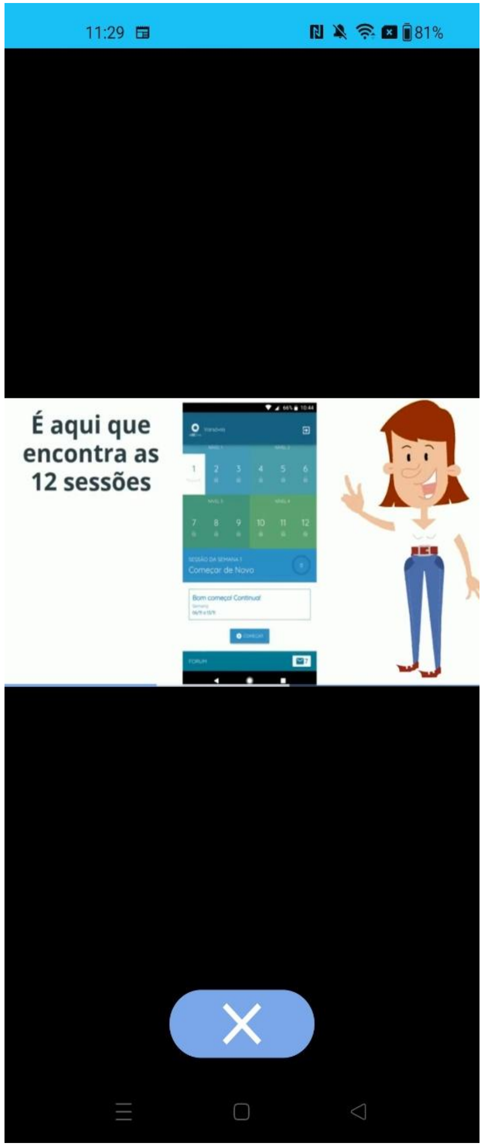
11:29
81%
É aqui que encontra as 12 sessões

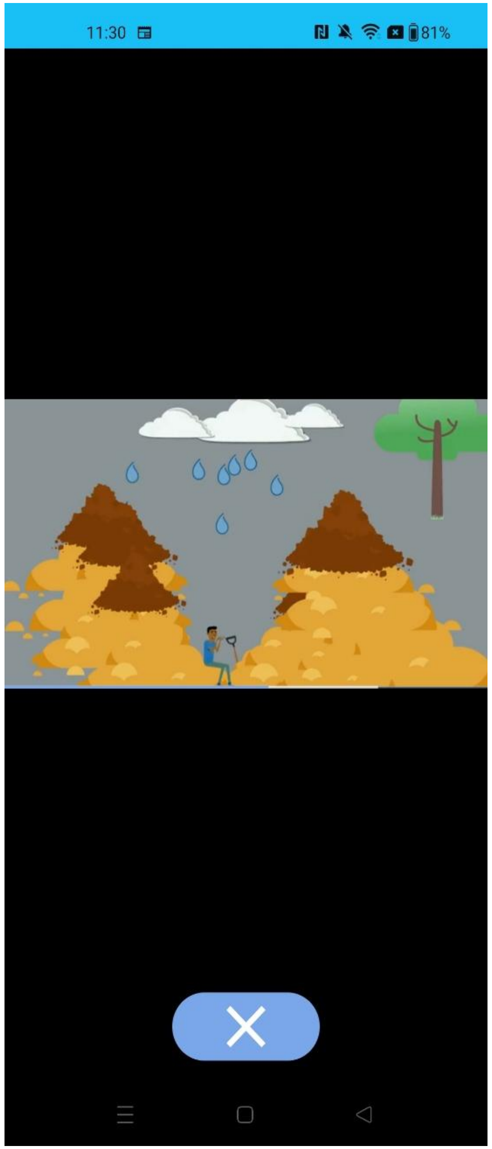
11:30
81%

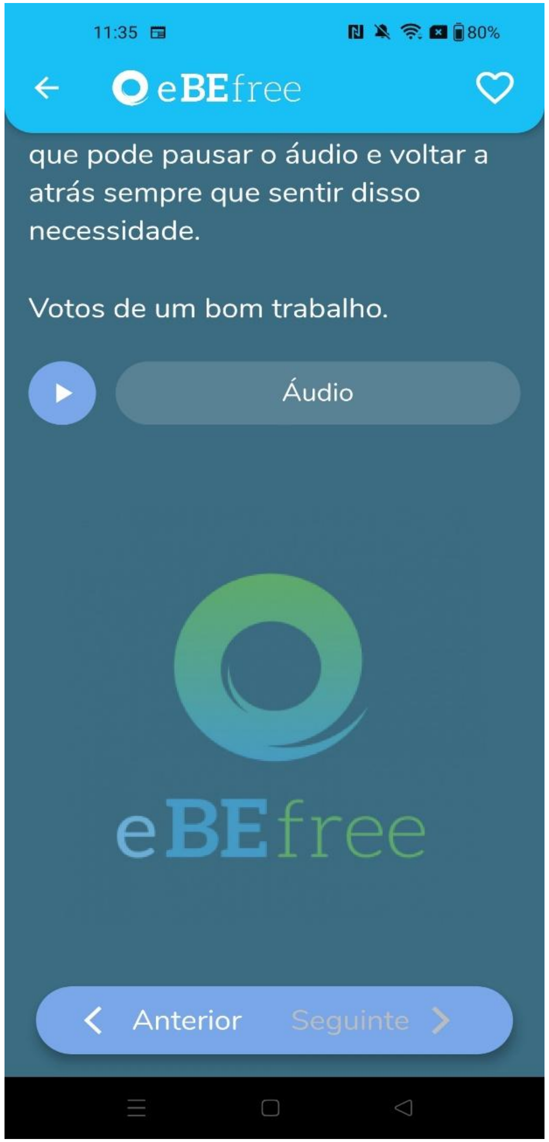
11:35
80%
eBEfree
que pode pausar o áudio e voltar a atrás sempre que sentir disso necessidade.
Votos de um bom trabalho.
Áudio
eBEfree
Anterior
Seguinte

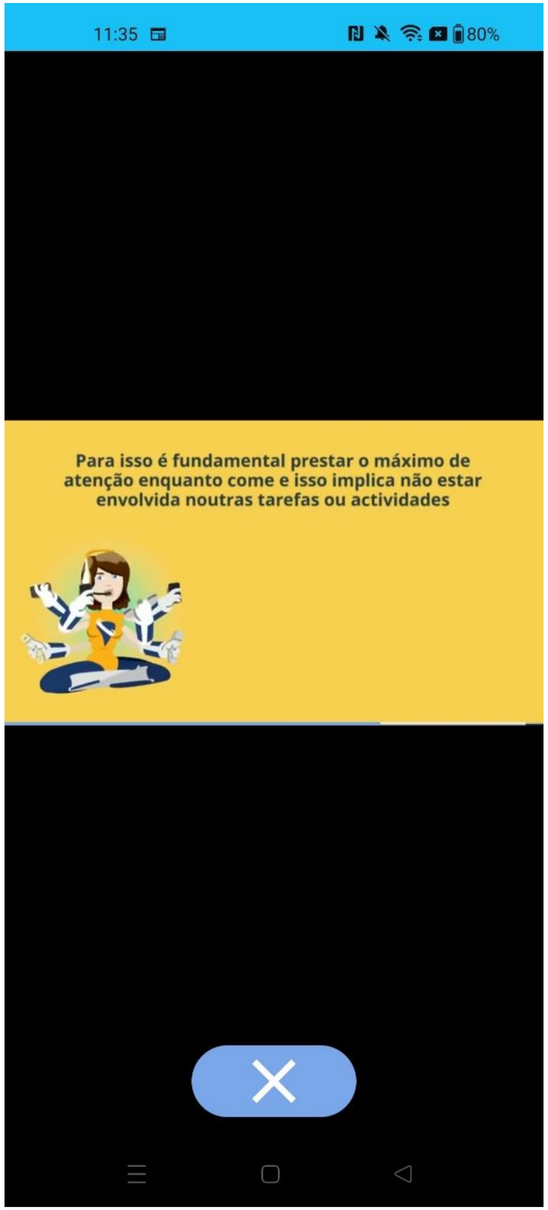
11:35
80%
Para isso é fundamental prestar o máximo de
atenção enquanto come e isso implica não estar
envolvida noutras tarefas ou actividades

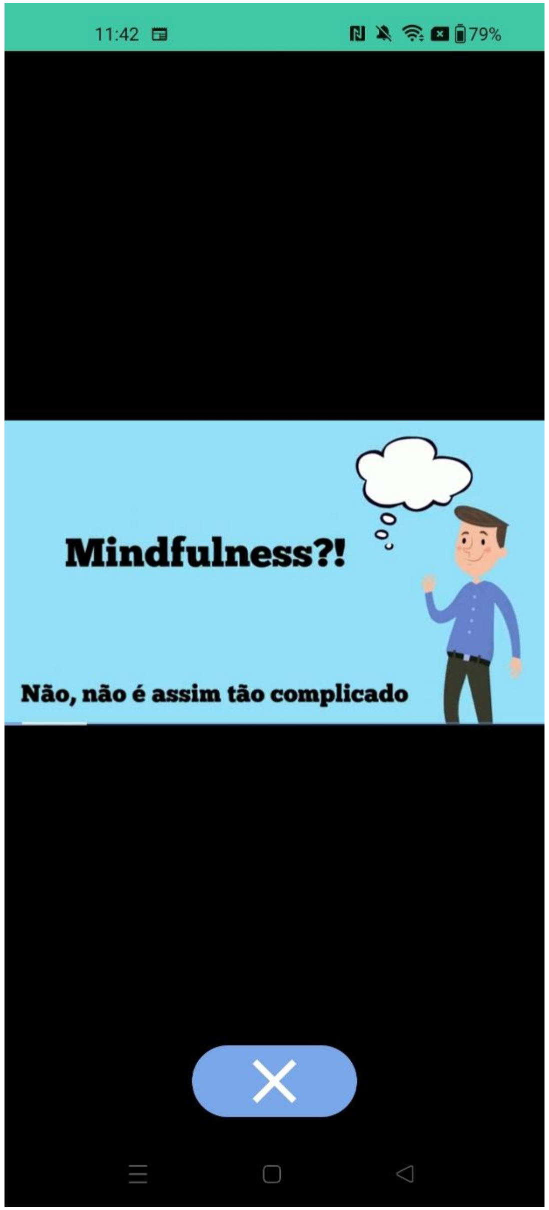
11:42
79%
Mindfulness?!
Não, não é assim tão complicado

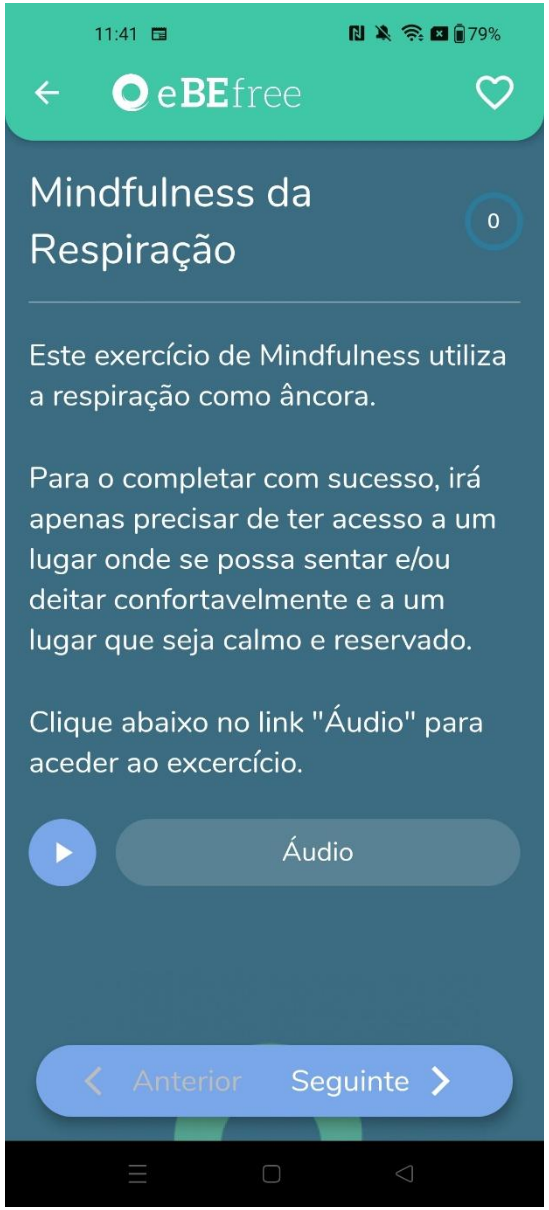
11:41
79%
eBEfree
Mindfulness da Respiração
0
Este exercício de Mindfulness utiliza a respiração como âncora.
Para o completar com sucesso, irá apenas precisar de ter acesso a um lugar onde se possa sentar e/ou deitar confortavelmente e a um lugar que seja calmo e reservado.
Clique abaixo no link "Áudio" para aceder ao exercício.
Áudio
Anterior
Seguinte

BAYESIAN HIERARCHICAL MODEL DIAGNOSTICS

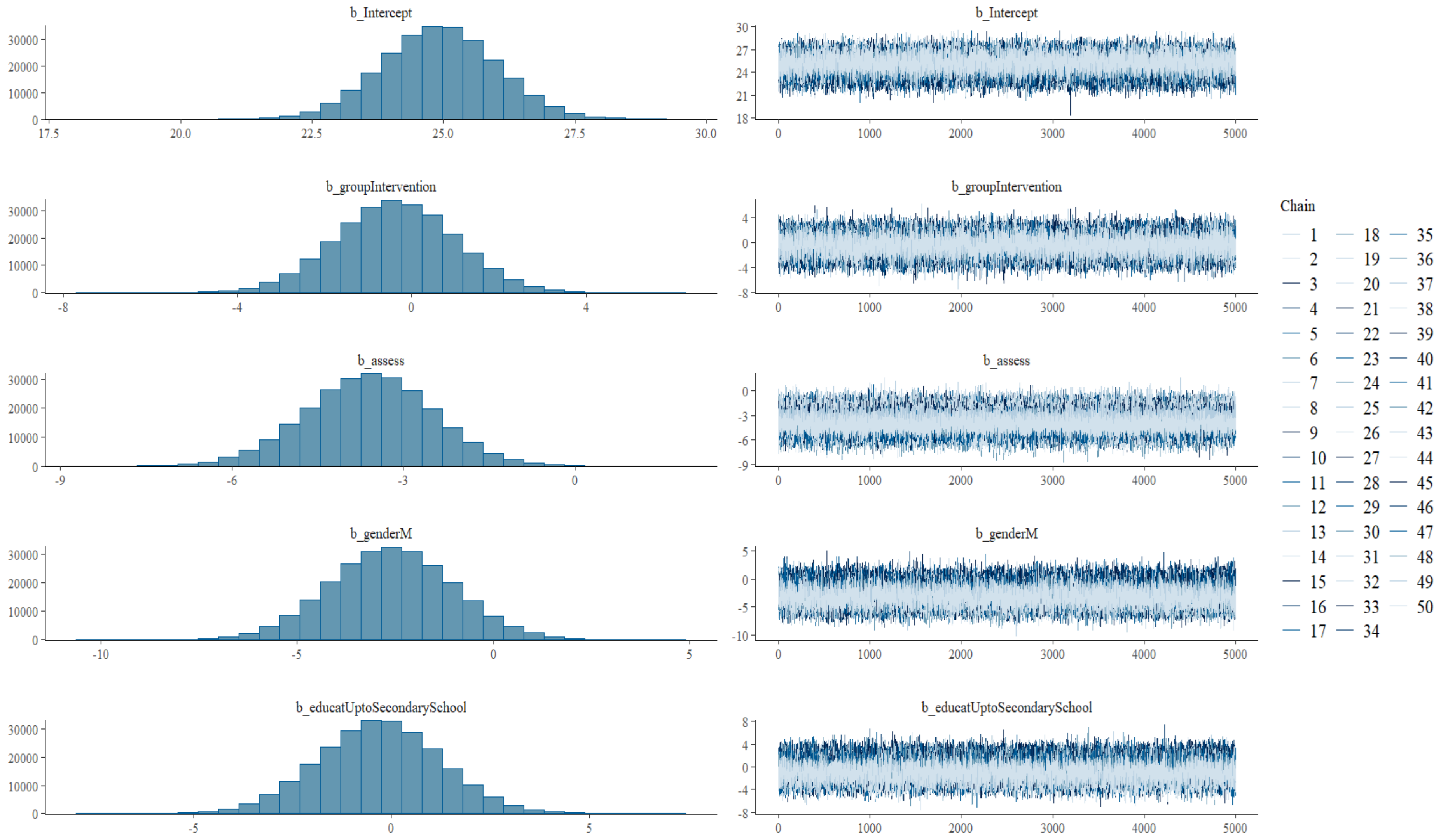

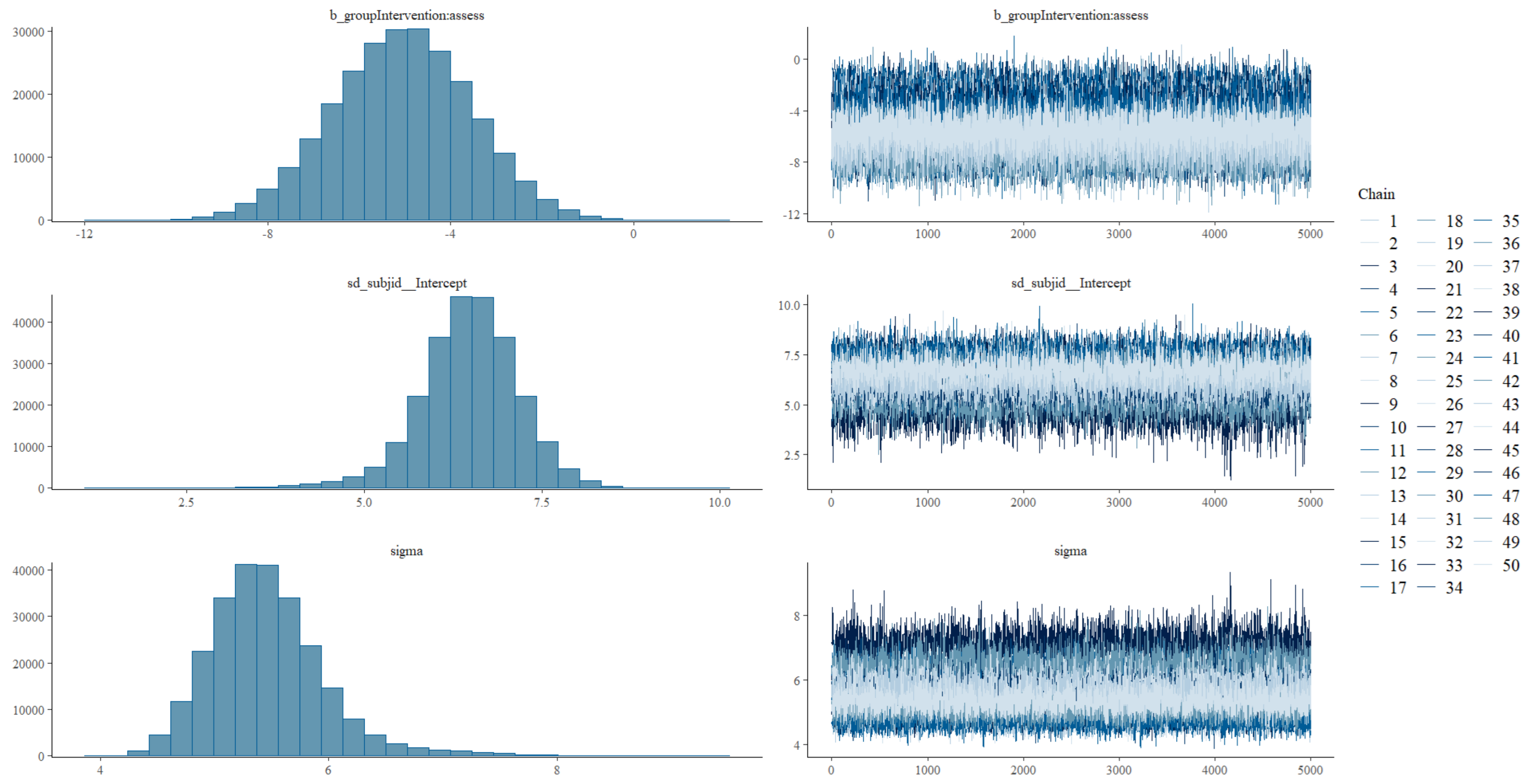

Figure A - Graphical representation of the posterior predictive density of model parameters suggesting normality of posterior distribution (left side), and Trace plots suggesting model convergence (right side).

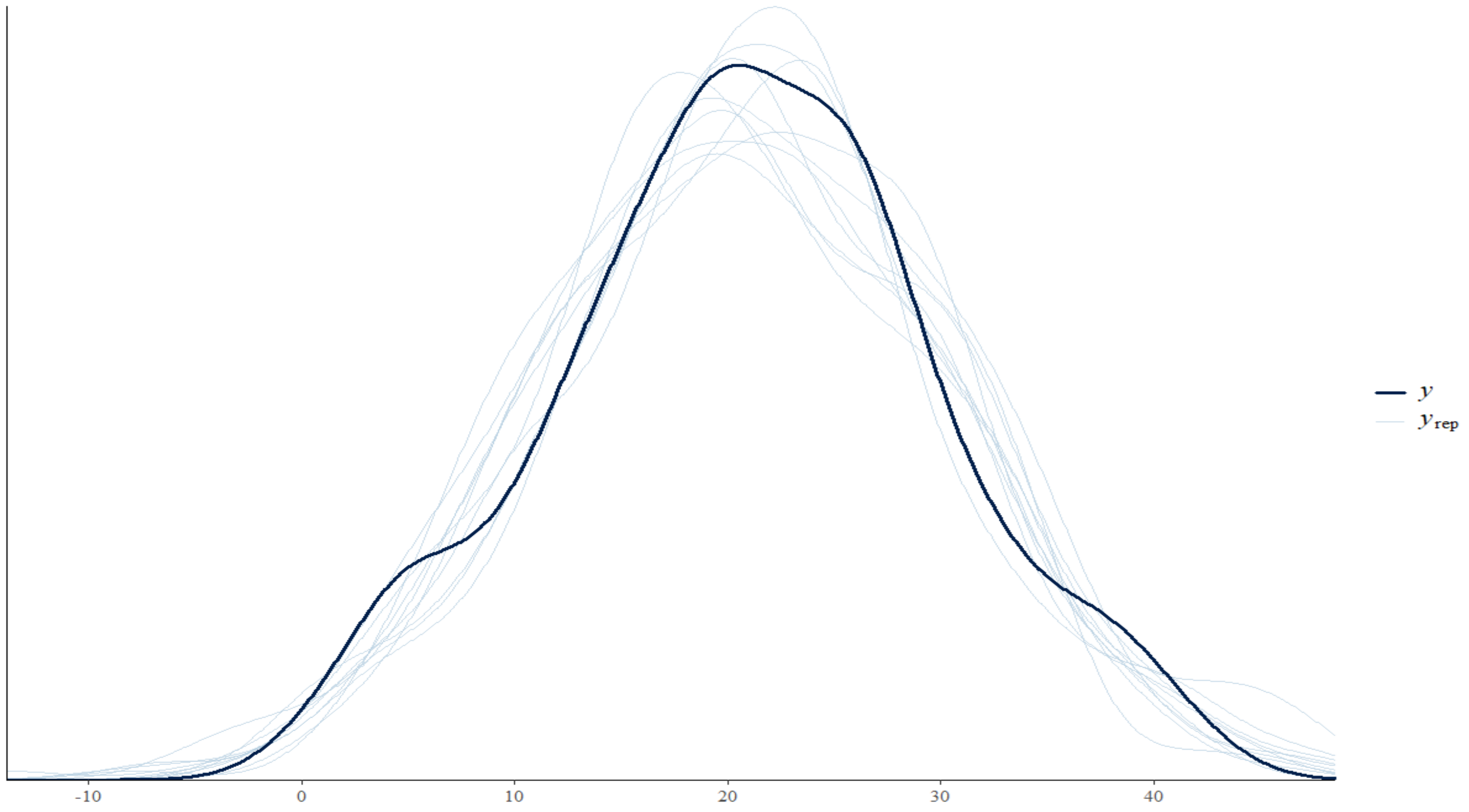

Figure B – Posterior Predictive Check Plot illustrating model convergence.
